# Coevolution of craton margins and interiors during continental break-up

**DOI:** 10.1038/s41586-024-07717-1

**Published:** 2024-08-07

**Authors:** Thomas M. Gernon, Thea K. Hincks, Sascha Brune, Jean Braun, Stephen M. Jones, Derek Keir, Alice Cunningham, Anne Glerum

**Affiliations:** 1 School of Ocean & Earth Science, University of Southampton, Southampton, UK; 2 Helmholtz Centre Potsdam – GFZ German Research Centre for Geosciences, Potsdam, Germany; 3 University of Potsdam, Potsdam, Germany; 4 School of Geography, Earth and Environmental Sciences, University of Birmingham, Birmingham, UK; 5 Dipartimento di Scienze della Terra, Universita degli Studi di Firenze, Florence, Italy

**Keywords:** Geodynamics, Geology, Geomorphology, Tectonics

## Abstract

Many cratonic continental fragments dispersed during the rifting and break-up of Gondwana are bound by steep topographic landforms known as ‘great escarpments’^1–4^, which rim elevated plateaus in the craton interior^5,6^. In terms of formation, escarpments and plateaus are traditionally considered distinct owing to their spatial separation, occasionally spanning more than a thousand kilometres. Here we integrate geological observations, statistical analysis, geodynamic simulations and landscape-evolution models to develop a physical model that mechanistically links both phenomena to continental rifting. Escarpments primarily initiate at rift-border faults and slowly retreat at about 1 km Myr^−1^ through headward erosion. Simultaneously, rifting generates convective instabilities in the mantle^7–10^ that migrate cratonward at a faster rate of about 15–20 km Myr^−1^ along the lithospheric root, progressively removing cratonic keels^11^, driving isostatic uplift of craton interiors and forming a stable, elevated plateau. This process forces a synchronized wave of denudation, documented in thermochronology studies, which persists for tens of millions of years and migrates across the craton at a comparable or slower pace. We interpret the observed sequence of rifting, escarpment formation and exhumation of craton interiors as an evolving record of geodynamic mantle processes tied to continental break-up, upending the prevailing notion of cratons as geologically stable terrains.

## Abstract (web summary)

By integrating geological observations, statistical analysis, geodynamic simulations and landscape-evolution models, a physical model is proposed to link the coevolution of craton margins and interiors with continental rifting.

## Main

**Fig. 1:**
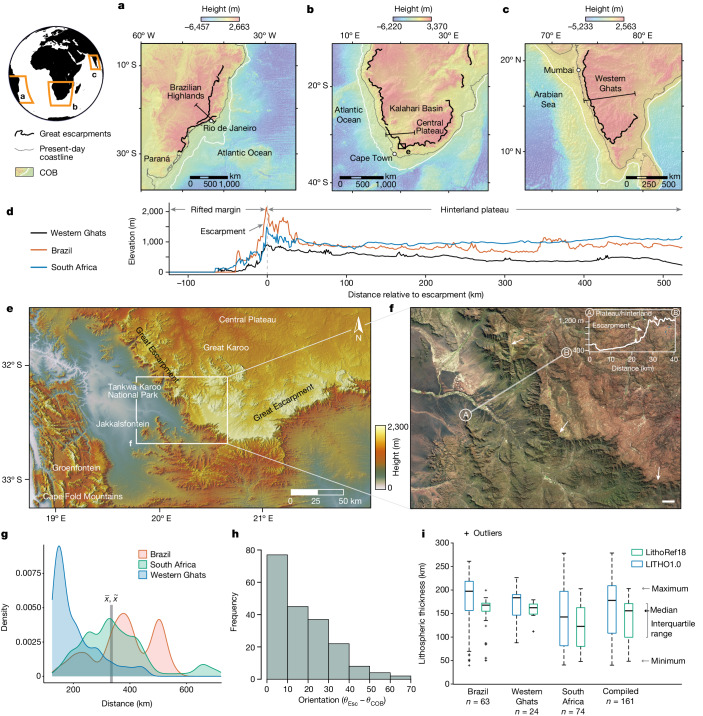
Location and physical characteristics of great escarpments. Global terrain maps for ocean and land (gridded data from GEBCO) of the east coast of Brazil (**a**), Southern Africa (**b**) and the Western Ghats (Sahyadri Hills), India (**c**) (see inset map for locations). The maps show a simplified representation of escarpments mapped using digital terrain models (Methods) and COBs from GPlates^59^ (https://www.gplates.org/). **d**, Topographic profiles of escarpments (see **a**–**c** for lines of section). **e**, Map of the Great Escarpment of South Africa (see **b** for location) generated using NASA SRTM elevation data (Lambert conformal conic projection). **f**, Short-wave infrared satellite image of the same escarpment (white arrows) from Sentinel Hub. A typical topographic profile is shown (A–B). Sinuosity of escarpments is related to contrasted retreat rates of channels relative to interfluves^1^. Scale bar, 5 km. **g**, Probability density for nearest distance between escarpments and COBs for the regions (see **a**–**c**). Global mean and median thicknesses (vertical grey band) are 336 and 333 km, respectively (*n* = 5,288; Extended Data Fig. 3a–c). Two distinct peaks for Brazil reflect two phases of escarpment formation there (Cretaceous and Cenozoic)^3^. **h**, Difference in orientation between escarpments (*θ*_Esc_) and COBs (*θ*_COB_) calculated using the perpendicular to the escarpment tangent at 50-km intervals (*n* = 195; Methods and Extended Data Fig. 2). Escarpments are typically sub-parallel to adjacent COBs (Extended Data Fig. 3d–g). **i**, Box plot of lithospheric thickness for each escarpment, point sampled from maps generated using LITHO1.0 (ref. ^41^) (blue boxes) and LithoRef18 (ref. ^42^) (green boxes) at 1.0°, or approximately 111-km, intervals (*n* = 161). Source Data

Cratons experience extremely low erosion rates when viewed over geological time^12^, a feature attributed to their mechanical strength and prolonged stability^13,14^. Thus, the formation of great escarpments (hereafter, escarpments) (Fig. 1) and subsequent uplift of craton interiors are geologically abrupt and enigmatic^15^ events that disrupt^6^ this long-term stability. Escarpments, that is, laterally extensive breaks in slope about a kilometre high and many thousands of kilometres long, typically occur near the edges of shields—tectonically stable regions rooted on strong cratons^16^ (for example, Eastern Brazil, Southern Africa and the Western Ghats of India; Fig. 1a–c). Although a widely held view is that these landforms originate during continental rifting^2–4,17–19^, the mechanistic linkages are not well resolved.

Escarpments have been attributed to various processes, including: (1) flexurally induced uplift along rift flanks owing to lithospheric unloading during extension^2,20–22^; (2) small-scale convection induced by lateral temperature gradients, driving uplift of rift shoulders^7,8^; and (3) downgrading of the coastal area and inland base-level fall^23^. Crucially, the relationship of these processes to anomalous exhumation that occurs in remote hinterland plateau regions long after rift termination^5,18,24,25^ is poorly understood. Flexural uplift is typically confined to the rift flanks and cannot explain the formation of an elevated continental interior. Studies have variously invoked prolonged plateau uplift^26–29^ (for example, through compression-induced uplift unrelated to rifting and break-up^28^), post-rift tectonic reactivation^29–31^ and passive-margin rejuvenation^17^. However, whether the last process occurs is disputed^3^.

Surface processes occurring far from rift zones^5,18,24,25,29^ (>500 km away) and long (tens of millions of years) after rift cessation seemingly prohibit a first-order role for rifting. Several studies, such as for the classic Great Escarpment of South Africa (Fig. 1d–f), instead propose that plate movement over a large, low-shear-velocity province, exposing the continent to deep, buoyant mantle upwelling, drives rapid uplift and surface erosion over a broader region^32^. However, such a superswell is not observed in dynamic support histories derived from diverse continental and oceanic records^33^. Further, evidence for protracted plateau uplift since escarpment formation^18^ contradicts the notion of post-break-up tectonic stability proposed by the downgrading model^23^. Alternatively, enduring surface uplift surrounding the escarpment might reflect intermediate-scale (approximately 1,000 km) present-day mantle convective support^16,22,32–34^, possibly associated with cratonic-edge-driven convection^17,35^. Stochastic inversion models indicate that dynamic mantle support contributes approximately 650 m to the regional elevation in Southern Africa, with the remaining elevation (about 670 m) attributed to the isostatic lithospheric contribution^34^. This estimate is supported by independent modelling studies that suggest up to 1 km of dynamic/static mantle support^15^.

Drawing from the above examples, the broader context of landform formation following continental break-up is heavily debated. This study aims to quantify the spatial and temporal relationships between rift systems and the generation of escarpments and plateaus, while using geodynamic and landscape-evolution modelling to gain a quantitative understanding of the mechanisms influencing these regions.

## Origin of great escarpments

**Extended Data Fig. 1:**
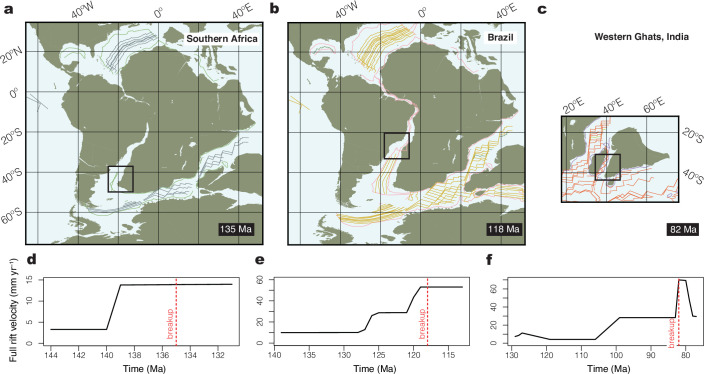
Timing and characteristics of Gondwana break-up in the studied cratons. Plate-tectonic reconstructions (from GPlates^59^), at the point of continental break-up, of Southern Africa at 135 Ma (**a**), Brazil at 118 Ma (**b**) and the Western Ghats, India, at 82 Ma (**c**). The plots in **d**–**f** show full-rift velocities for the corresponding rift systems (data from ref. ^54^), with the point of continental break-up labelled (dashed red line).

We begin by evaluating the physical characteristics of escarpments and examining their spatial and temporal relationships to continental margins and high-elevation hinterland plateaus. First, we focus on the main coastal escarpments, associated with cratonic lithosphere, which formed between 150 and 70 million years ago (Ma) during the break-up of Gondwana (Extended Data Fig. 1). We compare three classic coastal escarpments in Southern Africa, Brazil and the Western Ghats, spanning distances of approximately 6,000, 3,000 and 2,000 km, respectively (Fig. 1a–c). These length scales allow us to analyse their lithospheric properties using global reference models with low resolution (100–200 km). Older escarpments in northwest Africa and the eastern USA, associated with protracted rifting and break-up of cratonic lithosphere in the Central Atlantic (between approximately 240 and 180 Ma), feature more subdued topography shaped by prolonged post-rift erosion^22^ and are not a direct focus of our study.

**Extended Data Fig. 2:**
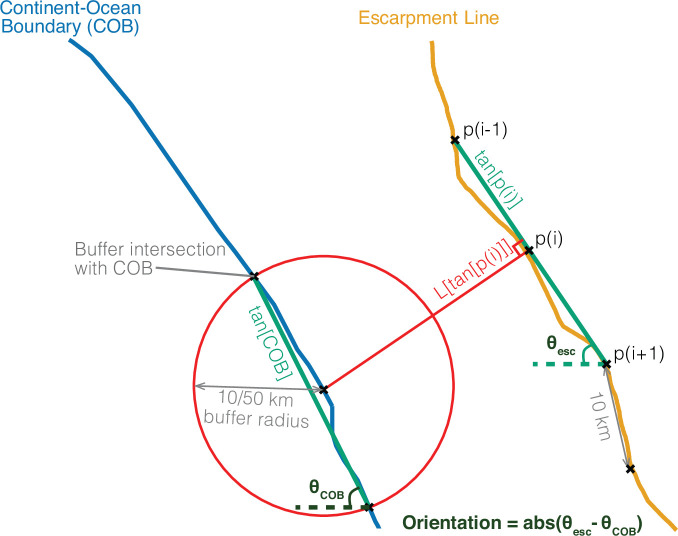
Defining spatial relationships between escarpments and COBs. Schematic showing how the relationship in orientation between COBs (blue) and escarpment lines (yellow) is derived in our analysis (see Methods for details). Note that the difference in orientation between COBs and escarpments is calculated as an absolute value to remove negative relative orientations.

Given their spatial and topographic characteristics, it is plausible that escarpments initiate as rift-border faults such as those kilometre-high escarpments separating the high-elevation Ethiopian Plateau from the East African Rift today^36^. In that case, their orientation and spacing with respect to continent–ocean boundaries (COBs), delineating ancient rift axes, should be broadly similar and closely mimic those generated in numerical models^19^. To examine this, we map the escarpments in detail (Fig. 1a–d) using geoprocessing tools in the ArcGIS software package. We then analyse the first-order spatial and topological attributes of escarpments and COBs (Fig. 1d and Extended Data Fig. 2) using the statistical computing package, R (https://www.r-project.org/; see Methods).

**Extended Data Fig. 3:**
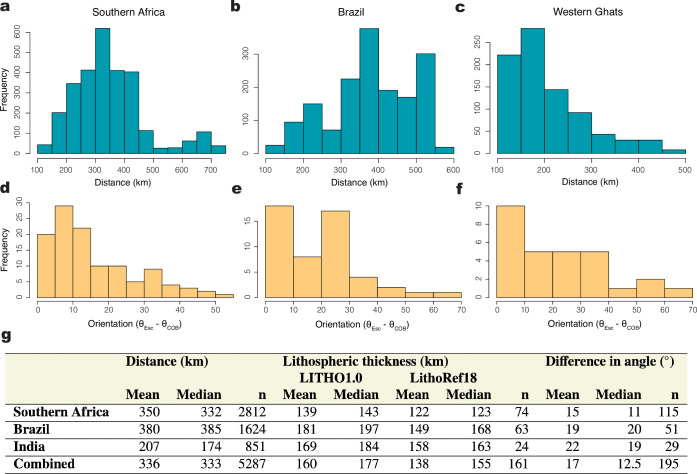
Physical relationships between escarpments and continental margins. Frequency distributions showing the nearest distances between great escarpments and COBs at 10-km intervals (Fig. 1a–c) for Southern Africa (**a**), Brazil (**b**) and the Western Ghats (**c**) (data summarized in panel **g**). **d**–**f**, Frequency distributions showing the absolute difference in angle between escarpments and COBs, calculated using the perpendicular to the escarpment tangent at 50-km intervals (Methods). **g**, Distance between escarpments and COBs, their associated lithospheric thicknesses from two global reference models (LITHO1.0 (ref. ^41^) and LithoRef18 (ref. ^42^)) and the absolute difference in angle between escarpments and the adjacent section (that is, perpendicular to escarpment tangents; see Extended Data Fig. 2) of COB at 50-km intervals (Methods).

The mean distance between escarpments and COBs varies across different regions, ranging from 207 km in the Western Ghats to 380 km in Brazil (Fig. 1g and Extended Data Fig. 3g). Global mean and median distances range from 330 to 340 km (Fig. 1g and Extended Data Fig. 3g). When we compare the orientations of geographic domains of escarpments to the adjacent sections of COBs (Methods), we find that they are sub-parallel over scales ranging from 10^2^ to 10^3^ km (Fig. 1h). These data suggest that escarpments originate at or near border faults, that is, at the inner boundary of rifted continental margins. Indeed, the distance between escarpments and the nearest oceanic crust (that is, outer boundary of the rifted margin) is similar to the estimated half-width of rift zones in the studied regions, which fall in the range 250–600 km (ref. ^37^). The mean distances between escarpments and COBs (Fig. 1g) closely align with predictions from numerical models^19^ and support rifting as a driver of escarpment formation^2–4^.

Rifting alone cannot satisfactorily explain the broad uplift and denudation patterns in hinterland regions, in which further scarp retreat occurred in the Cretaceous^5,18,24,25^. It is feasible that marginal uplift is more pronounced where the cratonic lithosphere is thick and underlain by a weak, basal layer that undergoes convective removal or delamination—a process that gives rise to isostatic uplift^38,39^. Since such processes are not expected to substantially thin the lithosphere (that is, more than about 35 km)^11,40^, the present-day lithospheric thickness offers a rough guide to that in the recent geological past. To investigate lithospheric-thickness characteristics along our coastal escarpments (Fig. 1a–c), we sample this property using two different global reference models—LITHO1.0 (ref. ^41^) and LithoRef18 (ref. ^42^)—at regular 1.0° intervals (commensurate with model resolution; Methods). Although locally variable, the escarpments generally occur on thick lithosphere, that is, the lithosphere–asthenosphere boundary (LAB) occurs at a median depth of 177 km or 155 km (Fig. 1i) for the two global reference models, respectively.

**Extended Data Fig. 4:**
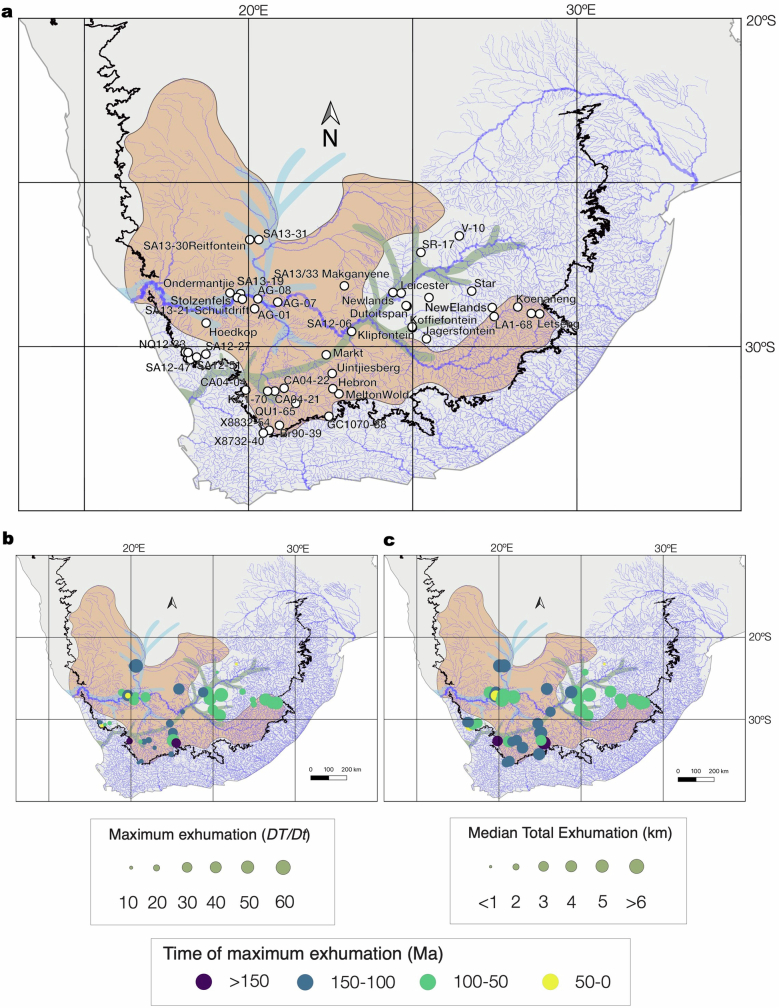
Fluvial systems across Southern Africa and the timing and location of Mesozoic exhumation. **a**, Present-day drainage regions in South Africa are shown, highlighting the Orange River drainage basin in orange (source: Department of Water Affairs, Republic of South Africa; https://www.dws.gov.za/iwqs/gis_data/river/All.html). The reconstructed footprint of Upper Cretaceous drainage systems—the Kalahari River (light blue) and the Karoo (or proto-Orange) River (green)—are based on ref. ^43^. Since the Upper Cretaceous, the mouth of the Orange River shifted north to its current location, merging with the Kalahari River. For context, the outline of the Great Escarpment (black line; see Methods) and locations of all studied thermochronology sites are shown (Extended Data Table 3). **b**, Time of maximum exhumation (coloured symbols) and rates maximum exhumation (d*T*/d*t*, proportional to the scale of symbols; see key) (Methods). Note that, in general, exhumation occurs earlier in the west and later in the east, where it is typically more intense (note also that younger exhumation in the west tends to be located near the Orange River or the Great Escarpment). **c**, Time of maximum exhumation (as in **b**) and median total exhumation (scaled; see key) for sites from 180 to 0 Ma.

The escarpments form primarily near the boundaries of continental lithosphere along rift-border faults (Fig. 1). Their sustained elevation is because of a combination of factors, including lithospheric thickness (Fig. 1i), flexural uplift^2,20,21^, mantle convection^7,8^ and dynamic mantle support^16,32–34^. Escarpments climb into higher terrain through headward erosion, causing them to retreat further inland^21^. This rapid retreat stops on reaching a point at which it functions as a pinned drainage divide, that is, a fixed boundary between drainage basins. For example, in response to tectonic uplift, the westward-draining Karoo River of South Africa (proto-Orange River; Extended Data Fig. 4a) incised a deep channel through the Great Escarpment^43^ at 120–110 Ma (refs. ^29,43^), paving the way for fluvial erosion of the hinterland plateau^44^. Because eroded sediments are largely transported westward into the Orange Basin, this phase of onshore denudation—a west-to-east ‘wave’ of erosion^44^—is recorded as a step increase in sediment accumulation rates in marine archives^29,45,46^. Drainage systems, which shaped plateau evolution, may have fundamentally responded to mantle processes (for example, delamination)^5,11,25,40,47^ following break-up. However, the nature of these geodynamic processes and their connections to geomorphology remain poorly understood.

## Modelling mantle–surface connections

**Fig. 2:**
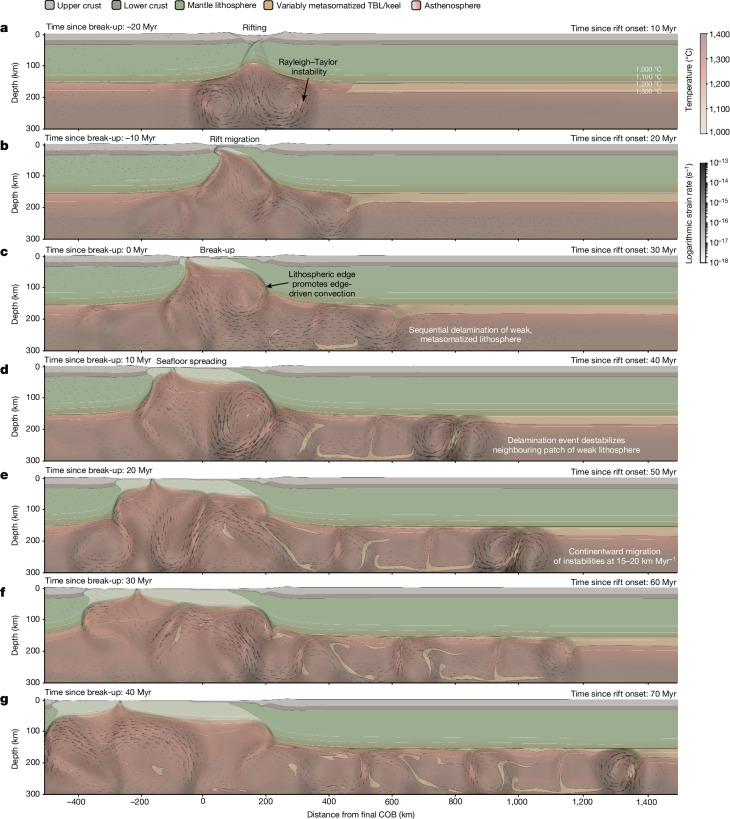
Geodynamic models of rift evolution. **a**–**g**, The sequential migration of Rayleigh–Taylor instabilities along the lithospheric keel, causing convective removal of the TBL (beige). This process, migrating at a rate of 15–20 km Myr^−1^, drives a ‘wave’ of isostatic uplift and surface denudation that similarly migrates across the craton at a comparable rate, and in some cases, more slowly, reflecting delayed landscape response times. The spatial and temporal extent of this process is limited by the width of the continent. Rift onset occurs 10 Myr before the time step shown in **a**, with continental break-up and seafloor spreading occurring in time steps **c** and **d**, respectively. Note that the reference frame is chosen such that the right continent is fixed, whereas the left continent is moving at 10 mm year^−1^. Values provided above each image on the left-hand side show timing relative to continental break-up in panel **c**. The images are adapted from ref. ^11^, which provides the animation for this reference model. In the simulations (see Supplementary Videos 1–4), the rift-border fault, or proto-escarpment, is 100–300 km from the COB.

Considering this gap, we investigate the influence of rifting and mantle dynamics on regional exhumation patterns. We use numerical thermomechanical simulations, building on our earlier work^11^ and applying conditions and material properties deemed reasonable within the context of previous geodynamic studies (Methods and Extended Data Table 1). Our simulations show that Rayleigh–Taylor (convective) instabilities^9,10^, with characteristic wavelengths of about 50–100 km, form at lithospheric edges beneath the rift^11^. The simulations show that instabilities are initiated by: (1) upward suction of low-viscosity mantle beneath the rifting lithosphere, causing the first delamination event (Fig. 2a); (2) formation of a lithospheric edge during continental necking, inducing lateral temperature and viscosity gradients that generate edge-driven convection cells (Fig. 2c); and (3) sequential delamination (Fig. 2c–g), which combines with (2) to produce complex, edge-driven convection patterns. Delamination exploits the density and strength contrast between the colder lithosphere and hotter asthenosphere across the thermal boundary layer (TBL)^11^. Instabilities migrate cratonward at a rate of 15–20 km Myr^−1^, sequentially removing the TBL to drive adiabatic upwelling of asthenosphere and kimberlite volcanism^11^ (Fig. 2).

**Extended Data Fig. 5:**
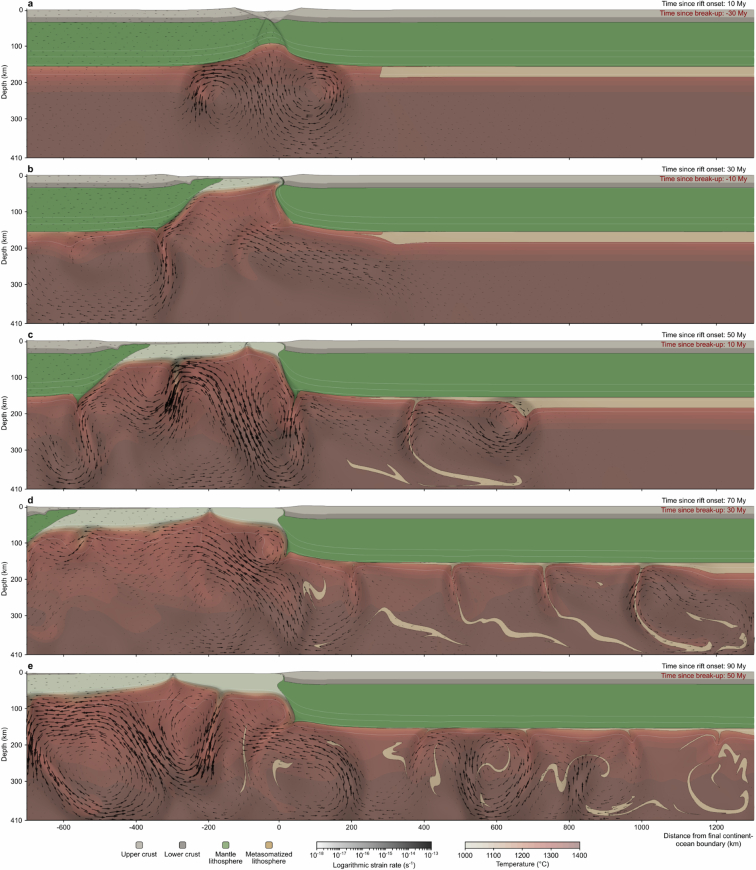
Geodynamic models of a 410-km-deep model domain. Frames from simulations showing different stages in the model evolution from 30 Myr before break-up (**a**) to 50 Myr after break-up (**e**). The depth of the model domain of 410 km was chosen to avoid complexities of the phase changes in the mantle transition zone. This model shows that sequential delamination occurs independent of the depth of the model domain. Instability migration velocity in this run varies slightly more than in the shallower scenario but averages 15–20 km Myr^−1^, identical to the reference model (see Fig. 2). Compared with the reference model (300 km deep), the 410-km model exhibits convection cells roughly twice as high; however, at the depth of the metasomatized layer, the horizontal distance between instabilities is very similar (about 250 km).

Although our reference model is 300 km deep and is pulled on one side only (Fig. 2), we further assessed the impact of symmetric boundary conditions, different extension velocities (5 and 20 mm year^−1^ instead of 10 mm year^−1^) and the vertical extent of the model domain (to 410 km; Methods and Extended Data Fig. 5). In all scenarios, the process of sequential delamination occurs as in the reference model, with no marked change in instability spacing (Supplementary Videos 1–4). Migration rates differ only slightly from the reference model (that is, 11 to 15 km Myr^−1^ for the symmetric model) and are in full agreement with observational constraints.

**Extended Data Fig. 6:**
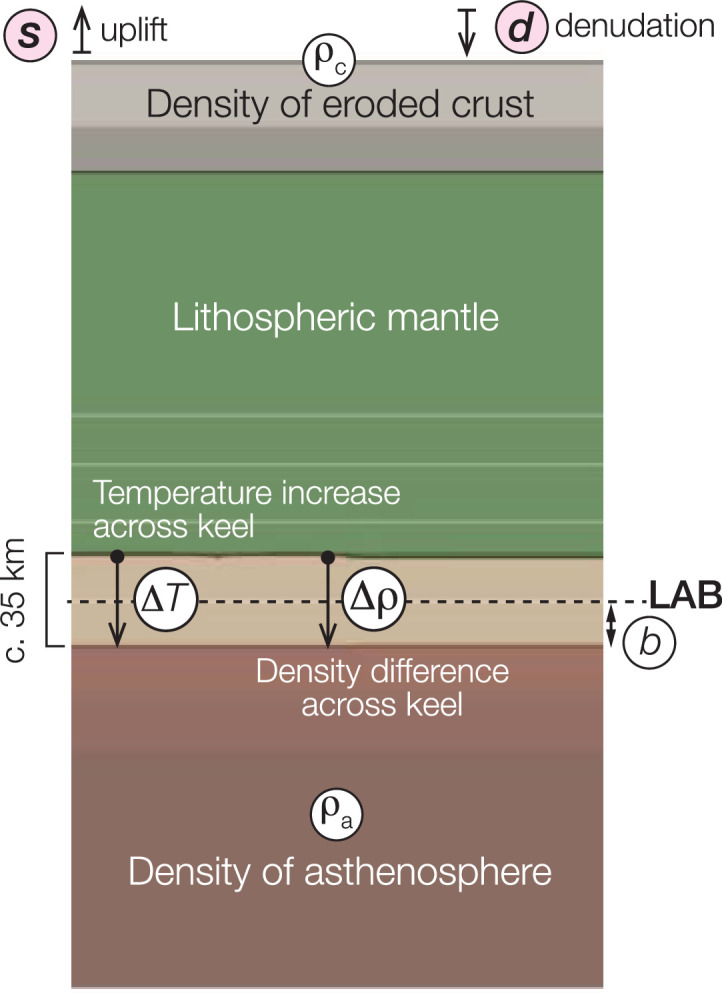
Main parameters used in the analytical models. Schematic showing the parameters used in equations (1)–(3) and defined in Extended Data Table 2. The metasomatized lithospheric keel is shown in beige.

We next ask how much crustal exhumation could realistically be driven by lithospheric removal. Our simulations imply that the lithospheric keel is removed rapidly over distances of hundreds of kilometres parallel to the continental break-up boundary (Fig. 2). The area of removed keel subsequently propagates hundreds of kilometres inland of the break-up zone. Thus, the footprint of the region in which the lithosphere has been thinned by removing its keel is much greater than the elastic thickness of the lithosphere. Therefore, we can use a simple Airy isostatic case to estimate the magnitudes of surface uplift and erosion. Rapid thinning of the lithosphere causes initial uplift at Earth’s surface of:

1 $$s=b\frac{\Delta \rho }{{\rho }_{{\rm{a}}}}$$

in which *b* is the thickness of the lithospheric keel that has been removed, *ρ*_a_ is the density of the asthenosphere and Δ*ρ* is the mean density difference between the lithospheric keel and the asthenosphere (not to be confused with the density difference between the asthenosphere and the crust; see Extended Data Fig. 6 for a schematic).

It is well established that uplift drives intensified surface erosion^38^, leading to further isostatic rebound. For the endmember case in which the new surface uplift is eroded back to the original base level, the total amount of denudation is given by:

2 $$d=s\frac{{\rho }_{{\rm{a}}}}{{\rho }_{{\rm{a}}}-{\rho }_{{\rm{c}}}}$$

in which *ρ*_c_ is the density of the eroded crust (Extended Data Table 2). Combining these two expressions gives the maximum amount of denudation in terms of the thickness of lithospheric keel removed:

3 $$d=b\frac{\Delta \rho }{{\rho }_{{\rm{a}}}-{\rho }_{{\rm{c}}}}$$

**Extended Data Fig. 7:**
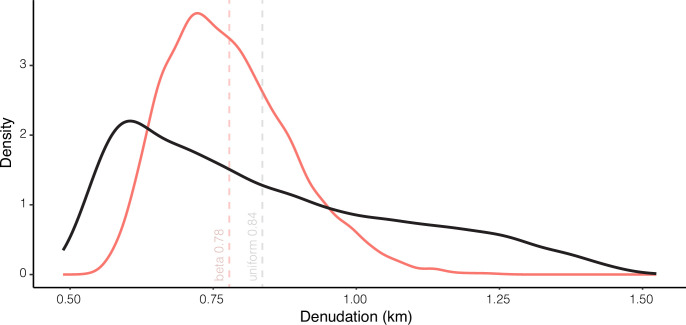
Likely denudation resulting from convective removal of the TBL. The plot shows the results of analytical models (equations (1)–(3); parameters given in Extended Data Table 2) used to estimate uplift and maximum denudation (Extended Data Fig. 6). The figure was plotted using a Monte Carlo simulation that sampled parameters from probability distributions, using both beta (red) and uniform (black) distributions (Methods).

Using indicative density values (Extended Data Table 2), the isostatic factor relating lithospheric keel thickness to initial surface uplift (equation (1)) is 0.003. The isostatic factor that relates initial uplift to maximum denudation (equation (3)) is 0.03–0.04; that is, the total erosion can be about an order of magnitude greater than the initial uplift. If the entire lithospheric TBL is removed, equivalent to removing an approximately 35-km-thick keel^11^, the initial uplift (equation (1)) will be about 50–100 m. This uplift can then be amplified by erosion (equation (3)), resulting in total denudation of approximately 0.8 km but, sampling for a range of variables (equations (1)–(3); Extended Data Table 2), plausibly lies in the range 0.5–1.6 km (Extended Data Fig. 7). Combining insights derived from our geodynamic simulations and analytical models, we anticipate that cratonic exhumation on a kilometre-scale should occur in the several tens of millions of years after break-up, and crucially, that the locus of denudation should progressively migrate inboard of escarpments over time (Fig. 2). Both predictions can be tested using surface-process constraints.

## Constraints from thermochronology

To examine the exhumation histories of cratons, we turn to thermochronology. Although much work has focused on escarpment retreat rates, which are relatively low (roughly 1 km Myr^−1^)^21^, we are primarily concerned with the long-wavelength patterns (covering distances of 10^2^–10^3^ km) of exhumation across cratons. We first compile published thermal history models, mainly from apatite (U–Th)/He (AHe) and apatite fission track (AFT) analyses (Extended Data Table 3) spanning a classic intracratonic region, the Central Plateau of South Africa^5,14,25,31,44,48–50^ (Fig. 1b and Extended Data Fig. 4). Many of these careful measurements were collected by Stanley et al.^5,25,44^, who compiled AFT and AHe ages for the region^18^ and documented a major phase of Cretaceous cooling across Southern Africa. The thermochronology data used in the original models, mostly derived in previous studies using the inverse modelling software HeFTy^51^, can potentially be reconciled by a field of viable time–temperature (*t*–*T*) paths. To estimate the most probable timing of cooling and evaluate uncertainties across 47 plateau sites, we use published best-fit *t*–*T* paths, upper and lower envelopes encompassing time uncertainty and individual model thermochronology curves (Extended Data Table 3). This information allows us to estimate the total temperature drop (°C), maximum rate of temperature drop (max(d*T*/d*t*), measured in °C Myr^−1^) and its corresponding timing (${t}_{\max }\frac{{\rm{d}}T}{{\rm{d}}t}$), along with associated model uncertainties. We assess max(d*T*/d*t*) over a 2-Myr symmetric (rectangular) moving window (see Methods).

**Extended Data Fig. 8:**
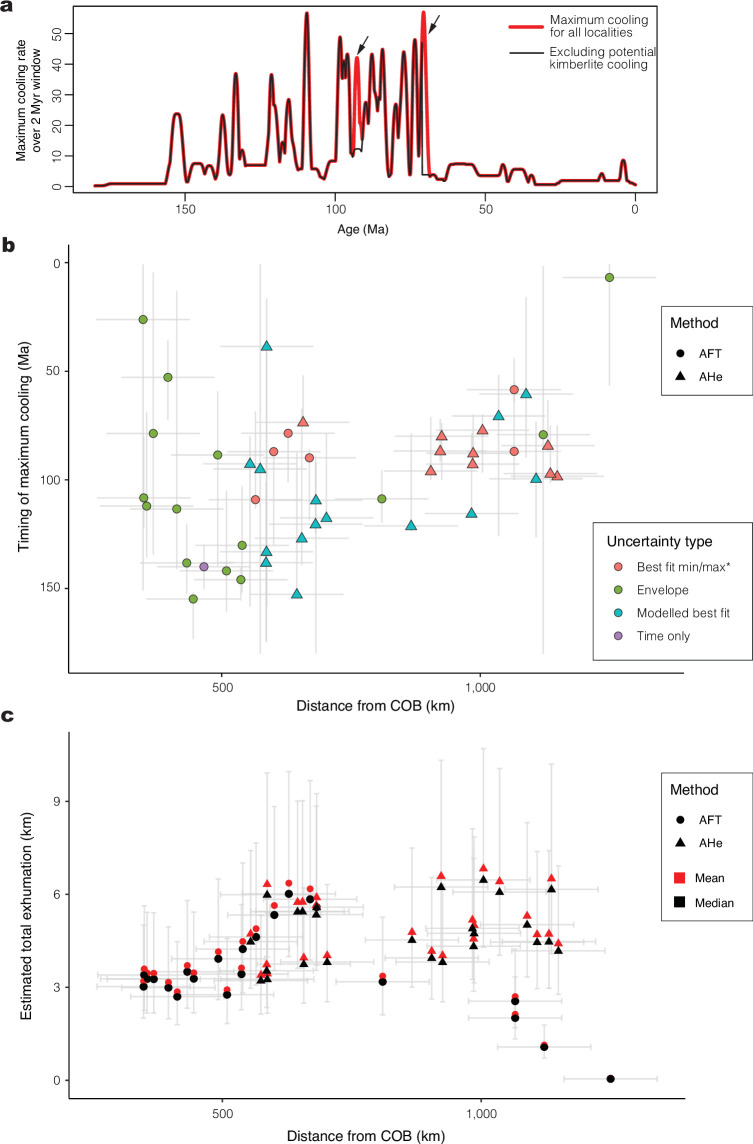
Estimated maximum cooling and total exhumation across the Central Plateau of Southern Africa. **a**, Maximum average cooling rate (d*T*/d*t*) over a 2-Myr rolling window for all localities in our analysis (see Extended Data Table 3), shown in red; the black curve shows the composite cooling curve, which removes all cooling events occurring near (that is, within 50 km) a kimberlite dated within ±2 Myr of the cooling event (see Methods). This effect potentially eliminates only two peaks (black arrows) and does not materially change the overall results. **b**, Timing of maximum cooling (*t*_mid_; Extended Data Table 3) versus distance from the COB (both variables with sampled uncertainty). *Note that ‘best fit min/max’ refers to cases in which a best-fit curve has minimum and maximum time estimates that relate to the limits of the field of ‘good fits’. **c**, Estimated total exhumation versus distance from the COB; this plot uses the best-fit total temperature drop and samples uncertainty in distance and geothermal gradient (Methods).

Our analysis confirms a protracted history of exhumation, with an acceleration in cooling between 120 and 110 Ma (Extended Data Fig. 8a), as recognized in previous studies^5,25^. This phase of exhumation coincides with a step change in kimberlite petrogenesis probably related to lithospheric removal^11,47^. In this region, denudation rates may have peaked at 175 m Myr^−1^ during the Cretaceous^48^. Such rates are anomalously high when viewed in the context of long-term cratonic erosion rates (about 2.5 m Myr^−1^) and high even with respect to young, high mountain belts^12^, making them difficult to explain in the absence of mantle forcing. The erosion was probably driven by an isostatic response to partial removal of lithospheric mantle^11,47^, supported by geochemical evidence for lithospheric delamination from deep kimberlite-hosted xenoliths^11,14,40,47^. Indeed, geothermometry of kimberlite xenoliths from the Kaapvaal Craton of South Africa indicate that upper-mantle temperatures were approximately 100 °C hotter after 100 Ma (ref. ^47^), pointing to profound thermal, chemical and petrological modification of cratonic lithosphere at this time^11,40,52^.

Although a component of the modelled cooling could be post-magmatic (that is, directly related to kimberlite volcanism), magmatic cooling is expected to be rapid (<10 kyr), in contrast to the longer-wavelength cooling trends observed (typically >2 Myr; Methods). Nevertheless, we evaluate this possibility by performing further modelling that conservatively removes all cooling spatiotemporally associated with kimberlites (Methods). This analysis confirms that magmatism can theoretically only explain a very small portion of the broad cooling trend observed over time (Extended Data Fig. 8a). The first-order trends are more consistent with denudation, as suggested previously^48^.

**Fig. 3:**
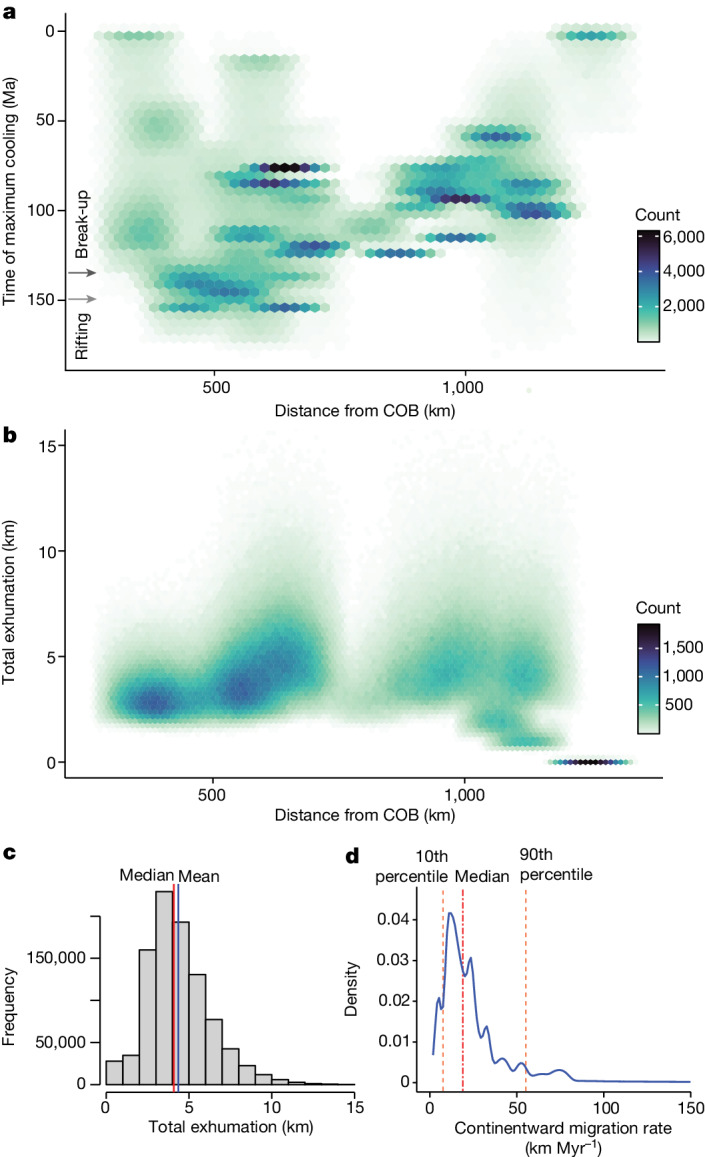
Exhumation of the Central Plateau of Southern Africa through time. **a**, Hexagonal heat map showing the statistically defined times of maximum temperature drop (${t}_{\max }\frac{{\rm{d}}T}{{\rm{d}}t}$) from thermochronology at 47 sites across the plateau (Extended Data Table 3). This plot was generated using Monte Carlo sampling (20,000 samples per site) of the estimated time and distance uncertainty at each site (see Methods and Extended Data Fig. 8b). **b**, Hexagonal heat map showing the estimated total exhumation for each site (47 sites, 20,000 samples per site) from 180 to 0 Ma using best-fit thermal history models and accounting for uncertainties in distance and geothermal gradient (see Methods and Extended Data Fig. 8c). **c**, Histogram of sampled total exhumation (*n* = 940,000), with mean and median values of 4.35 and 4.06 km, respectively. **d**, Density plot showing the apparent continentward migration rates of maximum cooling at thermochronology sites (*n* = 784,659 samples, or 87% out of 940,000; that is, post-break-up cases only) relative to the timing of continental break-up at the South Atlantic COB (135 Ma)^54^. The median rate is 19.1 km Myr^−1^ and the 10th and 90th percentiles are 7.7 and 55.2 km Myr^−1^, respectively. Source Data

**Extended Data Fig. 9:**
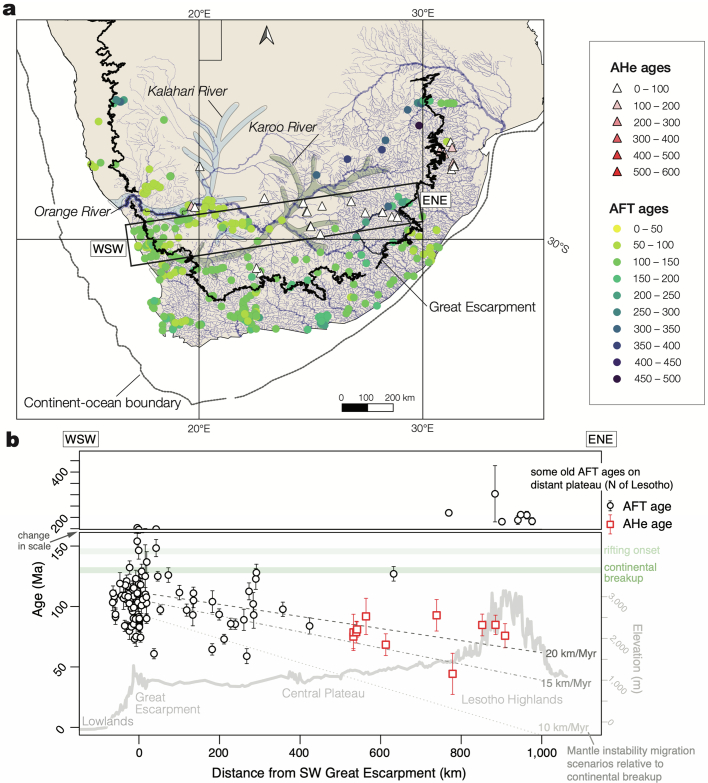
Distribution of AFT and AHe ages in Southern Africa. **a**, Map of Southern Africa showing the distribution of AFT and AHe ages (from ref. ^18^) relative to (palaeo)drainage basins and the Great Escarpment. Box shows the extent of the profile shown in **b**. **b**, Profile (see **a** for location) showing AFT (*n* = 99) and AHe (*n* = 11) ages^18^ from the southwest part of the Great Escarpment towards the interior (note the change in scale on the *y* axis at 150 Ma). Schematic elevation profile is shown for context as well as the timing of rifting onset and continental break-up. Dashed lines show the predicted timing of mantle disturbance considering instability migration rates of 10, 15 and 20 km Myr^−1^ (dashed lines) relative to the nearest COB at the point of break-up (that is, 135 Ma). The overall pattern shows good agreement with predictions from landscape-evolution models (Fig. 5).

Focusing on the Central Plateau, in which thermochronological data coverage is extensive (Extended Data Fig. 4), we examine spatial changes in exhumation through time (Fig. 3). Abrupt exhumation started near the South Atlantic margins during Upper Jurassic to Lower Cretaceous rifting^29,44,50,53^ and progressed eastward (Extended Data Fig. 4b), perhaps reflecting erosional scarp retreat processes^5,24,44^ (distinct from the earlier Great Escarpment that remained fixed near the plateau edge; Fig. 1). To further investigate this migration pattern, we analyse the timing of maximum cooling, ${t}_{\max }\frac{{\rm{d}}T}{{\rm{d}}t}$, across the plateau in relation to the distance from the South Atlantic rifted margins^54^. We find that, in general, the locus of uplift and denudation migrates inboard of continental margins after break-up (Fig. 3a and Extended Data Fig. 8b) and does so at a median rate of 19.1 km Myr^−1^ (Fig. 3d), overlapping with the migration rates of convective instabilities in our simulations (Fig. 2) and kimberlite volcanism^11^. This trend is consistent with a progressive decrease in AHe ages (using data from ref. ^18^) towards the east of the plateau (Extended Data Fig. 9).

**Extended Data Fig. 10:**
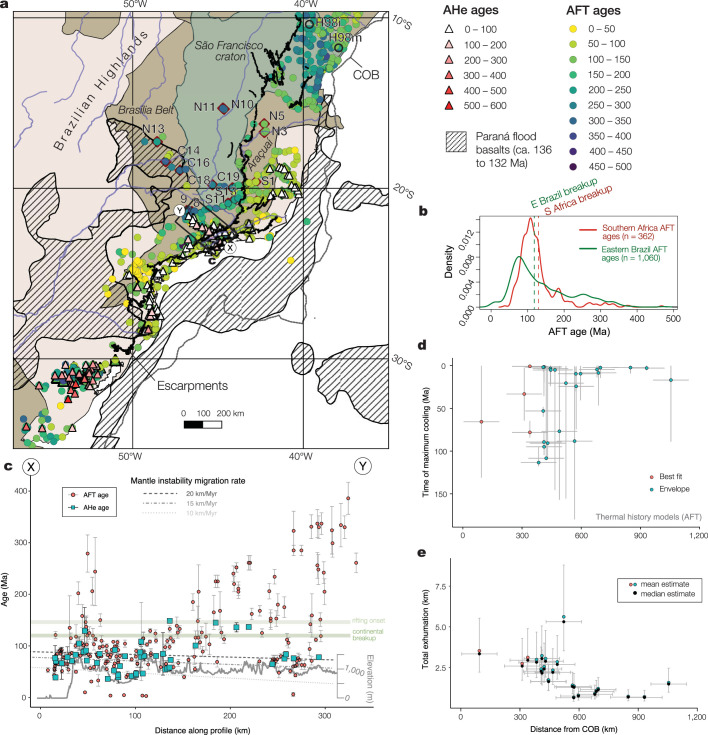
Timing and characteristics of exhumation in Eastern Brazil and AFT and AHe ages. **a**, Map of Eastern Brazil showing AFT and AHe ages (from ref. ^85^) and sites with thermal history models considered in our study (Methods). Spatial distribution of Paraná flood basalts is from ref. ^116^ and rivers are from Natural Earth (https://www.naturalearthdata.com/). **b**, Density plot comparing the distribution of AFT ages^18,85^ over time in relation to the timing of continental break-up in Eastern Brazil and Southern Africa (dashed vertical lines). **c**, Profile X–Y (see panel **a**) showing AFT (*n* = 226) and AHe (*n* = 45) ages^85^ from the continental margins towards the interior. An indicative elevation profile is shown, as well as the timing of rifting onset and continental break-up. Dashed lines show the predicted timing of peak mantle disturbance given  convective instability migration rates of 10, 15 and 20 km Myr^−1^ relative to the nearest COB at break-up (that is, 118 Ma). Time of maximum cooling (**d**) and estimated total exhumation (**e**) with distance from the COB using published thermal history models^26,30,56,57,111^ (*n* = 24). Note that **e** is based on the total temperature drop for best-fit curves, incorporating uncertainty in distance and geothermal gradient, with the error bars for exhumation signifying the 5th and 95th percentiles (see Methods for details). Note that the total exhumation is generally lower than that inferred for much of Southern Africa (Extended Data Fig. 8c), consistent with the observation that the Paraná flood basalts (shown in **a**) have been less eroded than their Etendeka counterpart.

Our analysis of thermal history models for Southern Africa^5,25,31,44,48,50,53,55^ indicates that the greatest plateau exhumation, averaging around 4 km in total since 180 Ma (sampling for uncertainty in geothermal gradients; Fig. 3b,c and Extended Data Figs. 4c and 8c), occurs for tens of millions of years after break-up, consistent with offshore sediment accumulation records^18,45,48^. In Southern Namibia, the escarpment and interior plateau show similar timing and magnitudes of exhumation relative to South Africa, starting in the lowlands and shifting into the continental interior tens of millions of years later (see Methods section ‘Testing broader applicability’). In Eastern Brazil (Fig. 1a), exhumation shifts from the continental margins (lowlands) during rifting and break-up—where it persists long after break-up—into the continental interior tens of millions of years later^56,57^ (Extended Data Fig. 10d). The available AHe ages show a general, though imperfect, trend towards younger ages cratonward, in good agreement with model predictions (Extended Data Fig. 10c). Although more work is needed to establish spatiotemporal variations related to rifting and break-up across the continents, these first-order patterns hint at a widespread process common to cratons.

## Landscape and geomorphic evolution

**Fig. 4:**
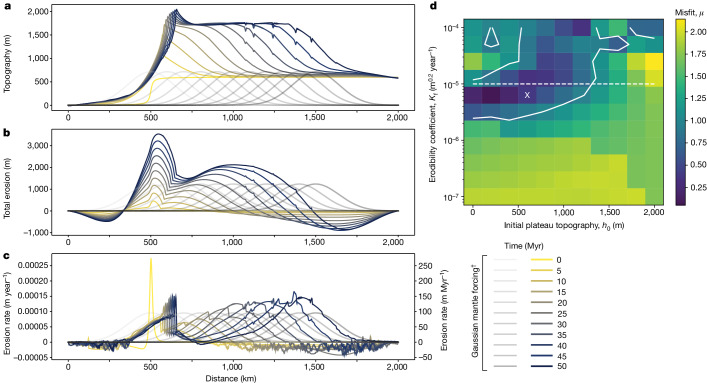
Landscape-evolution model. Model results showing the evolution of topography (**a**), total erosion (**b**) and erosion rate (**c**) over a period of 50 Myr following initial escarpment formation (Methods). Note how the escarpment becomes a pinned drainage divide, yet the broad locus of erosion migrates inboard in response to dynamic mantle (Fig. 2) forcing represented by the grey Gaussian curves (^†^*y*-axis scale not shown because of variations in the maximum uplift rate across model runs, which depends on the other parameters, namely, wave width, velocity, relative densities and initial topography; see Methods). **d**, Plot of the misfit function (*μ*) calculated by assuming optimum plateau height, denudation and final position of the drainage divide (Methods). Note that a misfit value < 1 means that these conditions are met and white contours indicate where this is true. ‘X’ denotes the position of the model (in parameter space) shown in **a**–**c**. Dashed line denotes the maximum limit of acceptable values for *K*_f_ that would allow the plateau to survive to the present day. Source Data

Rather than proposing a direct link between the rate of mantle convective removal and escarpment retreat, we suggest that a long-wavelength front of erosion migrates across the continent, tracking expected rates of convective removal. Although our geodynamic model cannot be used to directly interpret changes in surface topography, it can predict general areas of uplift and erosion over time (Fig. 2). To test whether our model is consistent with basic geomorphic principles, we thus use the well-tested landscape-evolution model Fastscape^58^. Fastscape is a ‘plan-view’ model that solves the stream power law (SPL) and flexure equations in 2D (*x* and *y* directions) to compute a vertical surface displacement and secondary quantities such as erosion, erosion rate and drainage area (for governing equations, see Methods). We first use this model to predict the topographic and erosional characteristics forced by a Gaussian-shaped wave of uplift that migrates laterally with a velocity of 20 km Myr^−1^ and that is characterized by a half-Gaussian width of 200 km, properties informed by simulations (Fig. 2). The model assumes an initial plateau topography of 500 m and an erodibility coefficient, *K*_f_, of 1 × 10^−5^ m^0.2^ year^−1^ (Methods). The model shows the predicted topography, total erosion and erosion rate at 5-Myr time steps over 50 Myr (Fig. 4a–c). Our preferred model falls in the range of plausible values for three key variables, namely, plateau height, total denudation and the final position of the drainage divide (Fig. 4 and Methods).

**Extended Data Fig. 11:**
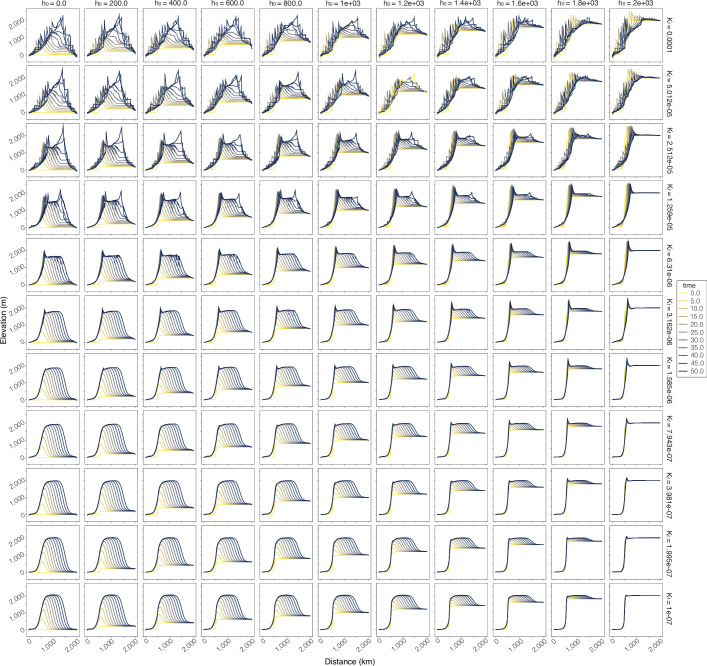
Plateau evolution scenarios in the surface-process model. Results of the landscape-evolution model showing how the topographic evolution of the plateau varies with different initial plateau topography values, *h*_0_ (shown along the top), and erodibility coefficient values, *K*_f_ (shown along the right-hand side). The key shows the different time steps in the model from 0 to 50 Myr.

**Extended Data Fig. 12:**
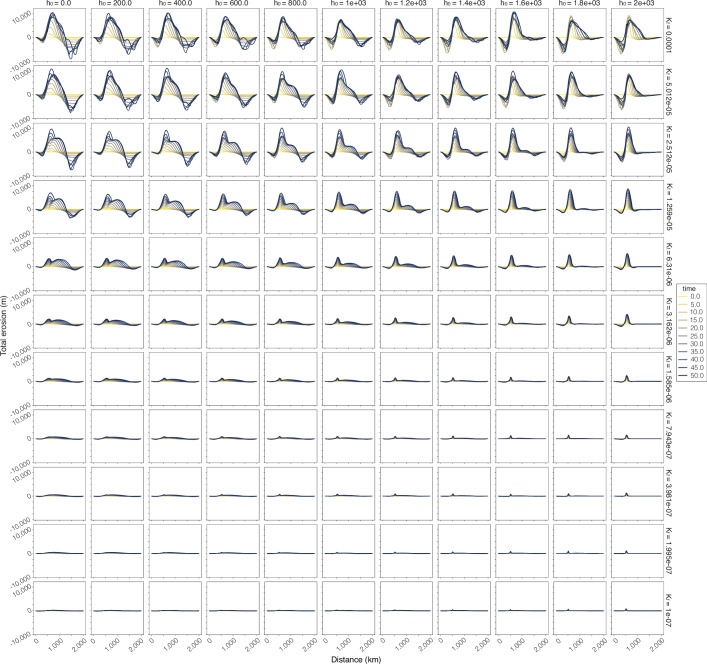
Erosion scenarios in the surface-process model. Results of the landscape-evolution model showing how the total erosion varies across the plateau over time in response to different initial plateau topography values, *h*_0_ (shown along the top), and erodibility coefficient values, *K*_f_ (shown along the right-hand side). The key shows the different time steps in the model from 0 to 50 Myr.

To explore the most probable range of *K*_f_ and initial plateau heights (*h*_0_), we perform 120 numerical experiments of landscape evolution (Extended Data Figs. 11 and 12) to calculate the misfit function (*μ*)—a mathematical function that measures the discrepancy between the modelled values of a system and the empirical observations of that system—assuming an optimal plateau height (1,650 ± 250 m), a total amount of denudation (2,750 ± 500 m) and the final position of the drainage divide (650 ± 100 km; Fig. 4d). Where the predicted values of the model fall between the assumed optimal value and uncertainty are shown in Fig. 4d, these suggest that a range of values for *h*_0_, between 0 and 1,000 m, provide a good fit to both observational constraints and models (Fig. 4). The models confirm that, although initially shaped by mantle processes near the rift, escarpments retreat inboard of the rift and stall at a relatively early stage to become a pinned drainage divide. Nevertheless, the broad wave of uplift and denudation continues to migrate towards the craton interior (Fig. 4b,c), as is generally observed (Fig. 3a). Further, the modelled pattern of erosion across the plateau (Fig. 4b) is in good agreement with observations (Fig. 3b).

**Fig. 5:**
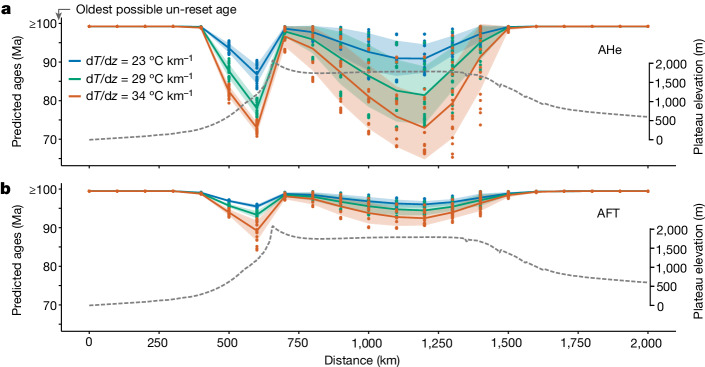
Predicted thermochronological ages in the surface-process model. Predicted ages from Fastscape for the AHe (**a**) and AFT (**b**) systems, assuming three different geothermal gradients and considering the same case as before, run for 50 Myr (that is, model ‘X’ in Fig. 4). Each point represents ages computed on a 21 × 21-point grid. The solid lines denote the mean (in the *y* direction) and the shaded areas correspond to the mean ± the standard deviation (in the *y* direction). For comparison with observed data in Southern Africa and Eastern Brazil, see Extended Data Figs. 9b and 10c, respectively. Source Data

Using Fastscape to compute predicted ages for AHe and AFT (Methods), we identify two distinct regions of AHe reset ages (Fig. 5): one along the escarpment related to continuing escarpment retreat (at which the youngest ages are typically found in South Africa^18^) and one on top of the plateau, with ages becoming younger from west to east in response to the eastward propagation of the mantle forcing (Fig. 4). AFT ages exhibit either minimal or no resetting on the plateau owing to the higher closure temperature of this system (Fig. 5). Consistently, thermochronology data indicate a wider range of AHe and AFT ages near escarpments (Fig. 3a and Extended Data Figs. 9 and 10). The models help explain cases at the northeastern fringes of the Central Plateau, at which either gradual or minimal exhumation occurred during the Mesozoic^53^—sites that fall outside the main catchment of the Karoo/Orange rivers (Extended Data Fig. 4a), constraining the probable northeastern limit of erosional propagation. Generally, the main patterns observed in our model (Fig. 5) show good agreement with data from Southern Africa and Brazil, with AHe ages being predominantly younger than AFT ages (Extended Data Figs. 9 and 10). Overall, this model serves as a useful benchmark to guide future thermochronological efforts in such regions.

## Deep Earth forcing of craton exhumation

Our findings show that a migrating wave of uplift (Fig. 3a–c), forced by sequential delamination of lithospheric keel, can generate a stable flat plateau even though thousands of metres of rock have been eroded (Fig. 4a–b). Estimated exhumation magnitudes from thermochronology spanning the past 180 Myr (Fig. 3b) are considered as upper-bound estimates, considering the partial preservation of Upper Cretaceous kimberlite pipes in Southern Africa^32^ that may imply bedrock erosion of 1.0–1.5 km, at least locally, since that time^24^. Although our analytical models suggest a maximum likely denudation of about 1.6 km due purely to delamination, these estimates do not account for uplift related to dynamic mantle support^34^, which may superpose to the vertical motion tied to convective delamination. Further, the analytical models do not consider density changes resulting from melt metasomatism in the lower lithosphere^11^ nor do they consider longer-term (that is, about 10 Myr) lithospheric thinning owing to convective removal. Geodynamic models suggest that such thinning^39^ can lead to extra uplift of more than 500 m.

Our model can explain the gradual eastward wave of exhumation across the Central Plateau^44^, which intensified during the Late Cretaceous (100–80 Ma; Fig. 3) and is consistent with plausible topography-formation scenarios^18,46^. Notably, peak exhumation rates (Extended Data Fig. 8a) coincide with the highest frequency of kimberlite eruptions after 100 Ma (ref. ^11^). Collectively, our current and previous work^11^ suggests that both processes are related to the sequential disruption of lithospheric keels (Figs. 2 and 5) but may operate on different timescales: kimberlite volcanism occurs rapidly, whereas uplift and denudation may (or may not) occur more slowly, reflecting slower landscape-response times that are modulated by regional differences in climatic and drainage conditions.

**Fig. 6:**
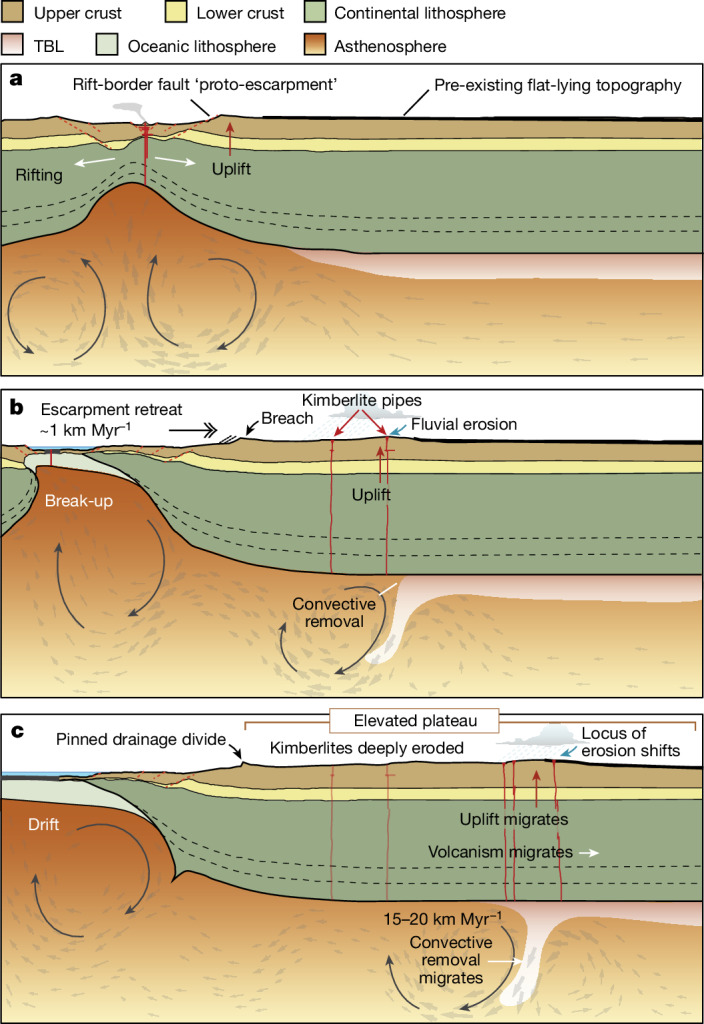
Simplified conceptual model of rifting, escarpment formation and exhumation of craton interiors. **a**, Rifting causes edge-driven convection in the mantle, rift-flank uplift and escarpment formation. **b**, Rayleigh–Taylor instability migrates along the lithospheric root, resulting in convective removal of the TBL of the lithospheric keel, driving kimberlite volcanism^11^, isostatic uplift and denudation. **c**, Escarpment becomes a pinned drainage divide that is locally breached by the main rivers draining the plateau. Meanwhile, the convective instability continues to migrate towards the continental interior, leading to isostatic uplift, a shift in the locus of erosion and plateau formation (Fig. 4).

We infer that the overall trend in exhumation magnitude, with two prominent clusters on the plateau (Fig. 3b), is controlled by the evolution of the fluvial landscape over tens of millions of years in response to delamination of lithospheric mantle keel (Figs. 4b and 6). While alternative topography-formation scenarios propose up to 1 km of plateau uplift in the Cenozoic^18^, global-scale landscape-evolution models—incorporating palaeoelevation and palaeoclimate forcings—simulate low erosion rates across the plateau during this time^46^, consistent with observations^53^. Even at the Cenozoic peak, sediment volumes in the Orange Basin were approximately an order of magnitude lower than those of the Late Cretaceous^45,46^, indicating much lower denudation during any late-stage plateau uplift. Although our model does not preclude a second plateau uplift phase during the Cenozoic, it favours primary topography formation during the Cretaceous. Nonetheless, prolonged low erosion rates over the Cenozoic shaped the present-day low-relief topography^43^.

## Surface record of continental break-up

Overall, our findings reveal a mechanistic linkage between continental rifting, escarpment formation and exhumation of craton interiors (Fig. 6), challenging the notion of ‘passive’ margins (supporting refs. ^30,50,55^). Our model identifies a common sequence of events during continental break-up, which includes: (1) rifting and escarpment formation; (2) full break-up and pinning of the escarpment; and (3) rift-driven delamination of lithospheric mantle, which sequentially migrates inboard of the rift zone (Fig. 2). Similarly, bedrock exhumation migrates sequentially, persisting for several tens of millions of years after rift cessation (Fig. 3). This exhumation, related to organized plateau growth (Fig. 4), offers a new explanation for the inferred secular exhumation of otherwise stable cratonic interiors^6^, explaining their enigmatic rejuvenation long after rifting and break-up (Fig. 6). Given that continental erosion strongly influences global chemical weathering intensity and palaeoclimates, this framework offers new insights into how the deep Earth regulates climate and biosphere evolution over geological timescales.

## Methods

### Mapping the great escarpments

We model escarpment features using the SRTM void-filled 15-arcsec GMTED2010 datasets digital elevation models (DEMs) from the United States Geological Survey (USGS). In the ESRI ArcMap 10.7.1 geodatabase, DEMs are mosaicked to produce composite raster DEM datasets, which we then use to map escarpments (in the World Geodetic System 1984 (WGS84) geographic coordinate system), using the Spatial Analyst toolset (slope, aspect and curvature). We then generate 100-m contours for the DEMs using the Spatial Analyst contour tool.

Triangulated Irregular Network (TIN) surfaces are generated using the Create TIN (3D Analyst) tool from the 100-m contour polyline datasets. TIN generation uses the Delaunay method of triangulation and WGS84 Transverse Mercator projected coordinate system. TIN surfaces are composed of mass points (TIN nodes), hulls and breaklines (hard and soft). The hard breaklines generated across TIN surfaces isolate breaks in slope that characterize escarpments. Isolation of hard breaklines (export as a separate polyline layer) is carried out using the TIN Line (3D Analyst) tool. We then use manual editing to isolate those breaklines that represent escarpments by alternating between slope, aspect, DEM and curvature base layers.

### Characterizing escarpment orientations

We next compare the orientation of discrete segments of escarpments and their associated COB (Extended Data Fig. 2). The mapped distributions of COBs are well established and described in key syntheses of plate-tectonic data^59,60^. However, we expect COBs to have some associated uncertainties, largely because these are zones rather than precise linear boundaries. For context, the global mean half-width of the COB ‘transition zone’ is approximately 90 km (ref. ^61^). As such, our distance analyses (Fig. 1g) described below is expected to carry uncertainties of ±90 km. In our analysis, we do not explicitly account for uncertainties in estimated distance to a given rift section, because these uncertainties are expected to be spatially correlated (that is, the same uncertainty value would apply to all points along a specific escarpment). COBs are exported from the open-source plate-tectonic software GPlates^59,60^ (https://www.gplates.org/). To enable this comparison, it is necessary to generate shapefiles for the escarpments with roughly equivalent complexity (degree of cartographic generalization) as the associated COBs. To achieve this, we use the open-source geographic information system applications QGIS (v3.16; https://www.qgis.org/) and GRASS (https://grass.osgeo.org/). Calculations are performed in the statistical computing package R (ref. ^62^; https://www.r-project.org/), using libraries sf (Simple Features)^63^, geosphere^64^, lwgeom and nngeo^65^.

We use a buffer to find the midline, or skeleton (simplified escarpment), in GRASS. This is done by: (1) converting each escarpment from WGS84 (EPSG:4326) to an appropriate projected coordinate system with units of metres, not degrees; (2) generating a (merged) 50/100/500-km buffer around each escarpment; (3) reducing by buffering again by −45/−99/−495 km; (4) applying the v.voronoi.skeleton function to compute the midline. These simplified shapefiles are then read into the R package to estimate the difference in tangent between the escarpment and continent boundary using the following procedure.

We read in these simplified escarpment and COB shapefile and define points (*p*(*i*)) every 10 or 50 km along each escarpment line. At each point, *p*(*i*), we then find the tangent, *Tp*(*i*), using the points either side (±10 or ±50 km) of the point of interest (Extended Data Fig. 2). We approximate the tangent by taking the line between *p*(*i* − 1) and *p*(*i* + 1). We then calculate the perpendicular to the tangent, *L*(*i*), and define the closest point of intersection, *x*(*i*), of line *L*(*i*) with the COB. Next, we calculate the tangent of the COB at *x*(*i*), *Tx*(*i*). We approximate *Tx*(*i*) by generating a 10-km or 50-km buffer around the point *x*(*i*). We then find the points of intersection of *Tx*(*i*) and the circular buffer (at which the boundary line enters and leaves the buffer) and use the line between these intersection points to estimate the angle. Finally, we calculate the difference (in degrees) between the tangents *Tp*(*i*) and *Tx*(*i*) and then the distance between points *p*(*i*) and *x*(*i*) (Fig. 1g–h, Extended Data Fig. 3).

### Analysing distances between escarpments and COBs

The escarpment polylines are exported from ArcGIS as shapefiles, matching the format of the COB files. We analyse the first-order spatial and topological attributes of both features using the R package, specifically, the sf^63^, lwgeom, nngeo^65^ and geosphere^64^ packages.

Nodes are defined at a spacing of every 10 km along both escarpment and COB polylines. The original coordinate reference system, GCS WGS84, is converted to appropriate EPSG codes for each region, allowing us to calculate distances in metres. Escarpment shapefiles are subsequently cleaned and converted to spatial features objects. Bounding boxes (buffer/crop) are defined for COB files, increasing the efficiency of searching for the closest point between escarpment and COB nodes. After cropping is complete, COB shapefiles are converted to spatial features objects. We use the dist2Line function to calculate the shortest distance in metres between escarpments and COBs (Fig. 1g and Extended Data Fig. 3a–c), with mean distances calculated using the ddply package^66^. Standard deviations are calculated for each plot using the sd function in the stats package in R.

### Lithospheric-thickness analysis

We analyse lithospheric-thickness distributions for the escarpments using two different global reference models, LITHO1.0 (ref. ^41^) and LithoRef18 (ref. ^42^). We perform surface interpolation from the vector points map by splines (0.1° cell size) using the GRASS function v.surf.rst. Next, we generate regular points along the length of escarpment shapefiles at 1.0° (approximately 110-km) intervals, using a QGIS vector geometry tool (points along a geometry). This chosen resolution is broadly commensurate with the resolution of the global models^41,42^. Using these specified sampling points, we then use the Point Sampling Tool in QGIS to obtain lithospheric-thickness values at each point from the interpolated raster map, and visualize the results for each escarpment using the boxplot function in Matlab R2021b (https://www.mathworks.com/products/matlab.html) (Fig. 1g). The range of lithospheric-thickness estimates for each escarpment using both of the above models are given in Extended Data Fig. 3g. It must be noted that LITHO1.0 returns what we suggest to be high estimates, being on average 10–15% higher than the corresponding LithoRef18 values (Extended Data Fig. 3g). This discrepancy probably arises because the density structure and geometry of boundaries in LITHO1.0 are not optimized to satisfy field data and lithospheric-thickness proxies, which may result in overestimation of the LAB beneath thick continental lithosphere^41^. Therefore, although we provide both measures for completeness, we consider the LithoRef18 values to give a more accurate picture of lithospheric thickness beneath the escarpments.

### Thermomechanical models

We use the finite element code ASPECT^67–69^ to compute the dynamic evolution of lithosphere and asthenosphere over a 100-Myr period. This geodynamic software solves the conservation equations of momentum, mass and energy for materials undergoing viscoplastic deformation^70^. We thereby use experimentally derived flow laws that account for temperature, pressure and strain-rate-dependent rheologies (Extended Data Table 1). The models are driven kinematically by prescribing velocity boundary conditions at lateral sides. The simulations generate a narrow rift that migrates laterally^71^, leading to a delay of lithospheric break-up. In agreement with previous work, pressure gradients beneath the rift induce pronounced rotational flow patterns^72^ within the asthenosphere. This flow destabilizes the base of the thermal lithosphere adjacent to the plate boundary, forming Rayleigh–Taylor instabilities that evolve self-consistently by sequential destabilization. Next, we describe the geometric and thermomechanical setup, along with the model limitations.

The domain of our reference model (Fig. 2) is 2,000 km wide and 300 km deep and consists of 800 and 120 elements in the horizontal and vertical directions, respectively. We chose a vertical model extent of 300 km depth to encompass the low-viscosity asthenospheric layer that is particularly prone to accommodating rapid mantle flow (an extent of 410 km is also tested). Although the lower boundary of this weak layer is not well defined, a model depth of 300 km includes: (1) the region beneath the lithosphere in which seismic anisotropy indicates a high degree of deformation (for example, ref. ^73^); (2) the depth range at which dislocation creep dominates deformation, leading to particularly low viscosity (for example, Fig. 10c in ref. ^74^); and (3) the highest depth at which carbonated melts can be expected to further reduce rock viscosity (for example, ref. ^75^). In our model, the initial distribution of material involves four homogeneous layers: 20-km-thick upper crust, 15-km-thick lower crust, 125-km-thick mantle lithosphere and 140-km-thick asthenosphere. To initiate rifting in a predefined area, we define a weak zone that features a 25-km-thick upper crust and 100-km-thick mantle lithosphere representing typical mobile belt conditions^41^. These layer thicknesses gradually transition to ambient lithosphere over about 200 km. For visualization purposes, we distinguish a 30-km-thick asthenospheric layer beneath some parts of the lithosphere as a simplified representation of metasomatized mantle.

The flow laws of each layer represent wet quartzite^76^, wet anorthite^77^, dry olivine^74^ and wet olivine^74^ for upper crust, lower crust, mantle lithosphere and asthenosphere, respectively (see Extended Data Table 1 for rheological and thermomechanical parameters). Our model involves frictional strain softening defined through a simplified piecewise linear function: (1) between brittle strain of 0 and 1, the friction coefficient is linearly reduced by a factor of 0.25; (2) for strains larger than 1, the friction coefficient remains constant at its weakened value. Viscous strain softening is included by linearly decreasing the viscosity derived from the ductile flow law by a factor of 0.25 between viscous strains 0 and 1.

In our reference model, we use velocity boundary conditions with a total extension rate of 10 mm y^−1^, equivalent to 10 km Myr^−1^. To test the sensitivity of our overall findings to this extension rate, we varied the extension velocity to slow (5 mm y^−1^) and fast (20 mm y^−1^) (Supplementary Videos 2 and 3, respectively). In these cases, we found that the process of sequential delamination still occurs as described in the reference model. Furthermore, we conducted two model runs to assess the influence of time-dependent extension velocities and verified that, in these cases, the key results remain unchanged. Material flux through the left boundary is balanced by a constant inflow through the bottom boundary. The top boundary features a free surface^69^. For simplicity, we fix the right-hand side of the model. However, we verified that our conclusions do not change substantially if extension velocities are distributed symmetrically at both side boundaries (Supplementary Video 1). For example, in this symmetric model, three instabilities are generated with migration rates of 20 and 16 km Myr^−1^ (when measured relative to the absolute reference frame) that translate to 15 and 11 km Myr^−1^, respectively, when accounting for rightward advection with 5 km Myr^−1^ (that is, in the reference frame of the continent). These values fit reasonably well to the reference model (15–20 km Myr^−1^; Fig. 2).

The surface and bottom temperatures are kept constant at 0 °C and 1,420 °C, respectively, whereas lateral boundaries are thermally isolated. The initial temperature distribution is analytically equilibrated along 1D columns before the start of the model by accounting for crustal radiogenic heat contribution, thermal diffusivity, heat capacity and thermal boundary conditions. We associate the bottom of the conductive lithosphere with the initial depth of the compositional LAB at a temperature of 1,350 °C. Below the lithosphere, the initial temperature increases adiabatically with depth. To smooth the initial thermal gradient between the lithosphere and the asthenosphere, we equilibrate the temperature distribution of the model for 30 Myr before the onset of extension.

The development of sequential instabilities and their migration velocity is a function of sublithospheric viscosity^11^. The occurrence of seismic anisotropy in the shallow asthenosphere suggests that deformation is dominated by dislocation creep^78^. We therefore use a nonlinear flow law using experimentally derived values for dislocation creep in olivine^74^, such as an activation energy of 480 kJ mol^−1^. Previous numerical models have shown that the occurrence of delamination is particularly controlled by the activation energy^79^, with a permissible range of 360–540 kJ mol^−1^. To explore the effect of activation energy on migrating instabilities, we conducted modelling experiments in which we varied the asthenospheric activation energy while keeping all other parameters constant. By decreasing the activation energy to 440 kJ mol^−1^, the viscosity of the shallow asthenosphere and metasomatized layer became roughly two times smaller. As a result, the lateral migration rates of the instabilities generated by this model were about twice as fast as in the reference model. The proportionality further agrees with estimates of migration speed from analytical considerations^11^. Rheological experiments as well as numerical and analytical modelling therefore indicate that the process of sequential delamination is plausible and that migration takes place at rates of tens of kilometres per million years—a speed that is comparable with the inferred wave of surface erosion within the plateau (Fig. 3).

Finally, we assessed the potential impact of the chosen model domain on our findings by increasing the depth to 410 km (Extended Data Fig. 5 and Supplementary Video 4). We chose this depth extent to avoid complexities associated with phase changes in the mantle transition zone. Most notably, this model shows that the process of sequential delamination occurs independently of the depth of the model domain. The migration velocity of the instabilities in this run is slightly more variable than in the shallower reference scenario but averages at a value of 15–20 km Myr^−1^, identical to that of the reference model. Notably, the spacing between two instabilities does not increase proportionally to the height of the convection cell. The 410-km model features convection cells approximately twice as high as in the reference model, whereas the distance between instabilities, when measured at the depth of the TBL, remains very similar (that is, the mean spacings for the reference model and 410-km model are 269 and 255 km, respectively). These observations lead us to conclude that, for the models to yield meaningful results, the TBL must be thin relative to the height of the convection cell—a criterion met in all cases described in our paper.

When interpreting our results, the following model limitations must be kept in mind. (1) We focus on first-order thermomechanical processes and do not explicitly account for chemical alterations, melt generation and magma ascent. (2) For simplicity, we assume that the initial depth of the LAB does not vary on the thousand-kilometre scale. We verified that gradual changes in morphology of the initial LAB did not affect our overall conclusions. (3) For simplicity, we neglect further processes in our generic modelling strategy that may be deriving from the impingement of mantle plumes, along-strike lithospheric heterogeneities and large-scale mantle-flow patterns.

### Analytical models and Monte Carlo simulations

We performed analytical modelling to estimate the magnitude of uplift and denudation resulting from the removal of the cratonic lithospheric keel, as described in ref. ^11^, using the parameters provided in Extended Data Table 2. It must be noted that this experiment considers only the density contrast between colder lithosphere and hotter asthenosphere^11^ and does not include compositional changes, for example, related to melt metasomatism. We assess the likely magnitude of erosion and denudation (equations (1)–(3)) by performing a Monte Carlo simulation, sampling parameters from probability distributions. We applied both uniform and beta distributions to represent natural variability in the parameters (Extended Data Table 2). For simplicity, we assume beta distributions with a standard deviation of 30% of the mean.

For the unstable TBL or keel, we considered a thickness (*b*) range of 17–18 km, as inferred from xenolith geotherm analysis^11^ (see Extended Data Fig. 6 for a schematic). This value represents half of the total thickness of the TBL, with the LAB situated near its middle. Similarly, the temperature increase across this layer, Δ*T*, is expected to lie in the range 140–165 °C, based on xenolith geotherm analysis^11^. Because our primary focus is on the southern Africa region, in which the most thermochronological constraints are available (Fig. 3), we used a range of densities of the eroded rock (*ρ*_c_) of 2,800–3,000 kg m^−3^ (ref. ^80^) to reflect a dominantly basaltic catchment in this region during the Cretaceous^5,25,48^. The uniform and beta distributions yield mean and maximum values for denudation of approximately 0.8 and 1.6 km, respectively (Extended Data Fig. 7). Over an extended time frame, that is, 10^6^–10^7^ years, dynamic mantle support will invariably increase this value.

### Thermochronological analysis

In our study, we test a geodynamic model (Fig. 2) by determining the spatial trends of, and total amount of, cooling (related to exhumation) over a specific interval, that is, 180–0 Ma. Because we are concerned with the exhumation history of cratons, we mainly restrict our study to those regions inboard of escarpments (that is, hinterland plateaus). We compile the thermal histories for a total of 47 sites across arguably the most classic example, the Central Plateau of Southern Africa (Extended Data Fig. 4). Details on the thermal models used in the original thermochronology work are provided in Extended Data Table 3. To estimate the most probable timing of cooling and evaluate uncertainties across the 47 plateau sites (Extended Data Table 3), we use published best-fit *t*–*T* paths, upper and lower envelopes encompassing time uncertainty, and individual model thermochronology curves (Extended Data Table 3). This information allows us to estimate the maximum temperature drop (max(d*T*/d*t*)) and its corresponding timing (${t}_{\max }\frac{{\rm{d}}T}{{\rm{d}}t}$), together with associated model uncertainties. Estimation of ${t}_{\max }\frac{{\rm{d}}T}{{\rm{d}}t}$ is not exact, particularly for sites that exhibit prolonged, gradual temperature change or with highly uncertain thermal histories. Different sources also estimate and present thermochronological uncertainty in different ways. Thus, for each site, we calculate a best estimate for ${t}_{\max }\frac{{\rm{d}}T}{{\rm{d}}t}$ (denoted *t*_mid_ in Extended Data Table 3), together with a range (that is, *t*_min_ and *t*_max_) accounting for available (published) model data and uncertainty estimates. These are shown as error bars in Extended Data Fig. 8 and are summarized in Extended Data Table 3. Using *t*_min_, *t*_mid_ and *t*_max_, we then fit a simple beta distribution to enable Monte Carlo sampling of the time uncertainty at each site (Fig. 3a,b). We apply the same approach to 24 sites from Eastern Brazil (Extended Data Figs. 10a). Here several sites (*n* = 4) show a distinct two-stage cooling history, which we accommodate in our analysis (Extended Data Fig. 10d).

For each thermochronology site, we interpolate to a regular (0.1-Myr resolution) time series over the period 180–0 Ma. We assume no (notable) temperature changes beyond the limits of the thermochronology data provided. We calculate the average temperature drop (d*T*/d*t*, in °C Myr^−1^) using a moving, symmetric window of ±0.9 Myr at each 0.1-Myr time step. The total temperature drop is calculated from the best-fit curves (d*T* total (bf) in Extended Data Table 3) and used to estimate the exhumation rate. Note there is no available temperature drop estimate for site Br90-39^81^ .

For all sites, *t*_mid_ is the best estimate of the timing of maximum temperature drop obtained from the best-fit curve for that locality. For sites at which we have upper, lower and best-fit curves, *t*_min_ and *t*_max_ are defined as the minimum and maximum time at which d*T*/d*t* ≥ 60% of max(d*T*/d*t*) over all three curves. Note that for some sites (for example, refs. ^29,50,53^), the upper and lower curves are described as defining the upper and lower 95% credible interval. For other sites (for example, ref. ^82^), the upper and lower curves define the ‘good-fit’ envelope.

Where we have a best-fit curve and further minimum/maximum time estimates, we define *t*_min_ as the earlier time of either the minimum estimate or the first point at which d*T*/d*t* ≥ 60% of max(d*T*/d*t*) on the best-fit curve. Similarly, *t*_max_ is defined as the later time of either the maximum estimate or the latest point at which d*T*/d*t* ≥ 60% of max(d*T*/d*t*).

For sites with several thermal history model runs, we calculate the earliest and latest times at which d*T*/d*t* ≥ 60% of the maximum over all model realizations, including ‘best’, ‘good’ and ‘acceptable’ model runs. For a given site, we define *t*_min_ as the 10th percentile minimum time and *t*_max_ as the 90th percentile maximum time estimate calculated from all model runs. The best estimate *t*_mid_ is defined as the time of the peak max(d*T*/d*t*) for the best-fit model run. For sites at which we do not have individual model runs, the best estimate *t*_mid_ is defined as the midpoint of the time interval at which d*T*/d*t* ≥ 90% of max(d*T*/d*t*) for the best-fit curve, to accommodate the lower resolution of these data. This approach gives uncertainty estimates that should be broadly comparable across localities.

We used the same published thermochronological constraints for 46 sites to estimate the total amount of exhumation at each site (Fig. 3b and Extended Data Table 3). Using best-fit curves for each site, we compute the maximum modelled temperature drop *T*_max_ − *T*_min_ over the interval 180–0 Ma. We then divide the temperature difference (*T*_max_ − *T*_min_) by the geothermal gradient, sampling from a beta distribution to capture the known uncertainty in the geothermal gradient. Geothermal gradients in Southern Africa today are estimated to range between 15 and 33 °C km^−1^ on average (ref. ^83^). Naturally, no single value of geothermal gradient can apply to the entire plateau. Hence, we consider a compilation spanning the present-day Southern African region^84^ to capture the plausible range. We represent uncertainty in the geothermal gradient by a beta distribution on the interval [10, 60] °C km^−1^ (informed by ref. ^84^) with a mean of 28 °C km^−1^, standard deviation 7.5 °C km^−1^ and parameters *α* = 3.3264 and *β* = 5.9136. The upper end of this range accounts for the very high values (38–46 °C km^−1^) favoured by Stanley et al.^18^ for Cretaceous Southern Africa. Distance is the shortest distance measured from the point location of the thermochronology site (longitude/latitude; Extended Data Table 3) to the COB line, calculated in R using the dist2Line function from the geosphere package^64^. Uncertainty in distance is again assumed to be ±90 km (see the section ‘Characterizing escarpment orientations’). We sample a distance offset using a beta distribution on the interval [−90, 90] km, with a mean of 0 km and standard deviation of 36 km, with parameters *α* = *β* = 2.625. In total, 20,000 samples are generated for both the geothermal gradient and distance offset for each thermochronology location (Fig. 3b).

Finally, we plot profiles of AFT and AHe ages across the Southern African (Extended Data Fig. 9) and Eastern Brazilian plateaus (Extended Data Fig. 10), using the data compilations of Stanley et al.^18^ and Novo et al.^85^, respectively. Here we constructed the profiles across the plateaus perpendicular to the continental margins and escarpments. In the case of Southern Africa, we avoided the southern part of the plateau in which the escarpment strikes roughly east–west, as it would cause interference (sampling parallel to an escarpment at which young ages are expected). In Eastern Brazil, we aligned our profile to capture the region at which most AHe ages exist and extend further inland. We include a buffer of 100 km to capture as many points as possible along a given section. In the case of Southern Africa, we plot the closest distance between the measurement point (AFT or AHe) and the escarpment at the western end of the profile (Extended Data Fig. 9). The distances were measured using the dist2Line function in the R package geosphere^64^, following the procedure outlined above for the escarpment analysis.

### Accounting for potential kimberlite-related cooling

The cooling detected by thermochronology studies can most parsimoniously be explained by denudation (for example, refs. ^5,24,25,48^). However, a component of the inferred cooling across Southern Africa (Fig. 3 and Extended Data Fig. 8) is feasibly related to magmatic cooling, for example (in the case of cratons), associated with kimberlite volcanism. We identify these potential cases to evaluate where the cooling is more likely to be denudational. Given the typical durations of cooling modelled in previous thermochronology investigations, these trends are unlikely to be driven by kimberlite magmatic cooling. Kimberlites are monogenetic volcanoes with probable eruption durations of hours or months^86^. Further, it has been shown that the largest kimberlite diatremes should cool down to ambient temperatures within 2–3 kyr of eruption onset^87^. Nevertheless, to explore whether such cooling could be important, we investigate all cases in which a kimberlite eruption age overlaps in time and space with the known locations of cooling (that is, thermochronology sites). In cases in which there was overlap, we completely (and conservatively) remove modelled cooling for a fixed time interval either side of the kimberlite radiometric age, using the kimberlite age compilation of Tappe et al.^88^. Specifically, we remove all cooling for sites at which there is a record of a kimberlite eruption dated within ±2 Myr (relative to the thermochronology time) and conservatively within a radius of 50 km, using thermochronology coordinates provided in Extended Data Table 3. Those purely denudational trends and those that account for potential kimberlite cooling are distinguished as red and black lines, respectively (Extended Data Fig. 8a). The above approach is considered conservative given the small length scales of kimberlite pipes (typically hundreds of metres) and that kimberlite eruptions (lasting on the order of several thousand years) and cooling of diatremes are known to be short-lived phenomena encompassing several thousand years at most (refs. ^86,87^).

Distances were estimated from longitude/latitude coordinates for the thermochronology sites and kimberlite records using the R geosphere^64^ distm function, using distGeo to obtain an accurate estimate of the geodesic, based on the WGS84 ellipsoid. Our analysis shows that even using conservative spatial and temporal bounds, cooling directly linked to kimberlite volcanism makes, if anything, a comparatively minor contribution to the overall cooling trends (Extended Data Fig. 8a). Notably, the period experiencing the most frequent (possible) kimberlite-related cooling between 100 and 80 Ma is associated with a marked increase in sediment accumulation rates offshore Southern Africa; previously, this event has been linked to a concomitant massive increase in onshore denudation^89^. Hence we argue that the observed cooling is largely denudational rather than magmatic (corroborating earlier suggestions^48,89^). We instead argue that the temporal coincidence between some kimberlites and modelled cooling is probably related to a common fundamental underlying mechanism (for example, lithospheric delamination), as opposed to a causal link between kimberlites and cooling.

### Landscape-evolution model

To investigate the evolution of topography, total erosion and erosion rate over time, we use the Fastscape landscape-evolution model, described in detail in ref. ^58^ (see ‘Code availability’). The model solves the SPL, which states that the rate of change of surface elevation, *h*, owing to river incision is proportional to local slope, *S*, and discharge, *ϕ*, put to some powers, *n* and *m*, respectively:

4 $$\frac{\partial h}{\partial t}=-K{\phi }^{m}{S}^{n}$$

This relationship can be traced back to the pioneering works of Gilbert^90^ and, from a computational point of view, Howard et al.^91^. Assuming that rainfall is relatively uniform (compared with slope and drainage area), the relationship is often simplified to yield:

5 $$\frac{\partial h}{\partial t}=-{K}_{{\rm{f}}}{A}^{m}{S}^{n}$$

the canonic form of the SPL, in which *A* is drainage area^92^. It is also known as the stream power incision model (SPIM) and its appropriateness at representing the main driver of landscape evolution in high-relief areas has been amply discussed^93^. In particular, much work has focused on the value of the exponents (*m* and *n*). It is unclear what the optimum values should be or on what they depend, but the ratio of the two, *m*/*n*, is close to 0.5 and can be derived from the concavity of river profiles. Here we used *n* = 1 and *m* = 0.4, as is commonly done. The method used to solve it is fully described in ref. ^58^. The other important equation that is solved is the biharmonic equation representing the elastic isostatic flexure of the lithosphere:

6 $$D({w}_{xxxx}+2{w}_{xxyy}+{w}_{yyyy})=({\rho }_{{\rm{a}}}-{\rho }_{{\rm{s}}})gw+{\rho }_{{\rm{s}}}g(h-{h}_{0})$$

in which *D* is the flexural rigidity, given by:

7 $$D=\frac{E{T}_{{\rm{e}}}^{3}}{12(1-{\nu }^{2})}$$

in which *E* is Young’s modulus, *T*_e_ the effective elastic plate thickness, *ν* Poisson’s ratio, *w* the deflection of the lithosphere caused by the difference in height, *h*, and a reference value *h*_0_, *g* the gravitational acceleration and *ρ*_s_ and *ρ*_a_ are the surface and asthenospheric densities, respectively. It is solved using the spectral method developed in ref. ^94^. The version of Fastscape used in our study has been used in many publications, including ref. ^95^.

For our purposes, the model captures the surface evolution of a continent over 50 Myr. The initial plateau topography (*h*_0_) was set to 500 m (considered broadly representative for Mesozoic South Africa^96^ and stable cratons globally^15^) and an erodibility coefficient, *K*_f_, was set to 1 × 10^−5^ m^1−2*m*^ per year, in which *m* is the area exponent in the SPL (*m* = 0.4). The topography and erosion characteristics were modelled using a Gaussian-shaped wave of uplift with a velocity of 20 km Myr^−1^ and half-Gaussian width of 200 km, with both properties informed by our thermomechanical simulations (Fig. 2). The amplitude was set to achieve 2,000 m of uplift after isostatic adjustment and taking into account the initial, pre-existing topography. This uplift wave mimics the dynamic topography generated by convective mantle flow. The maximum uplift rate varies from run to run, as it depends on all the other parameters, including wave width, velocity, relative densities and initial topography. For the ‘fixed’ parameters, that is, wave width, velocity and density ratio, the uplift rate varies linearly with the initial topography, with a range of approximately 1–0 mm year^−1^, as the assumed initial topography varies from 0 to 2,000 m.

The topographic response in the model includes a flexural isostatic response with a crustal density of 2,800 kg m^−3^ and asthenospheric density of 3,200 kg m^−3^, as well as an effective elastic thickness (*T*_e_) of 20 km. This thickness is considered an average value for continental lithosphere/crust^97^ (note that ref. ^97^ argues that values of *T*_e_ are often overestimated), which is further supported by the fact that it is not physically possible to ‘pin’ an escapement as a drainage divide if *T*_e_ is too high^21^. Any surface erosion in the model leads to further isostatic uplift, meaning that any change in topography related to erosion requires about 6–7 times the amount of erosion. This value is broadly in line with our independently derived analytical model (equations (1)–(3)) which predicts up to 10 times amplification of uplift by erosion.

In terms of boundary conditions, the left-hand and right-hand sides of the model have a fixed boundary at *h* = 0, representing the ‘base level’, which—in this case—corresponds to the ocean. The other boundary conditions are defined as periodic, meaning that a river flowing towards one of these boundaries (north or south) reappears on the other side of the model (south or north). This approach is commonly used when solving the SPL to avoid boundary effects. The routing algorithm^98^, which computes the direction of water flow, assumes that every drop of water falling on the model must eventually escape through one of the base-level boundaries. However, the specific path of escape is not predetermined; rather, it is internally computed from the local slopes and thus following the topographic evolution set by the uplift and fluvial erosion/carving. In the model results (Fig. 4), some water escapes through the left boundary and some escapes through the right boundary. Whether the escarpment becomes a divide and later evolves into a ‘pinned divide’ is not prescribed but instead results from a delicate balance between uplift and erosion. These points have been extensively discussed in the literature and are summarized in ref. ^21^.

We conducted a sensitivity analysis by assessing the range of values for *h*_0_ that would provide a good fit to observations. To do this, and to determine the quality of our model, we calculated a misfit function assuming that the optimum plateau height is 1,650 ± 250 m (in line with expectations^32,33^ and the present-day topography, which is 1.0–1.5 km on average^15^; Fig. 1d), optimum denudation is 2,750 ± 500 m (refs. ^5,25,29,31,48,50,53^) and the optimum final position of the divide is 650 ± 100 km (assuming that the wave started moving at 500 km from the left-hand side of the model). As we are concerned with the final position of the drainage divide, we use the upper end of our empirical estimates of escarpment position (Fig. 1g). We perform 120 numerical experiments of landscape evolution to calculate the misfit function (Fig. 4d and Extended Data Figs. 11 and 12). A misfit value less than 1 indicates that realistic conditions are met (that is, the model predicts values that fall between the optimum value ± the assumed uncertainty) (white contours in Fig. 4d). Our analysis also identifies the maximum limit of acceptable values for *K*_f_ that would allow the plateau to survive until today (Fig. 1d), without prohibitively high levels of erosion (dashed line in Fig. 4d). We find that the range of values for *h*_0_ that provide a good fit to existing constraints is from approximately 0 to 1,000 m. On this basis, our preferred model (Fig. 4a–c) has a misfit value less than 1 and obeys observational constraints.

### Predicting thermochronology ages in the surface-process model

To identify model limitations and guide future testing of our geodynamic model (for example, with improved spatial resolution of thermochronology studies and/or extra borehole data), we used Fastscape to predict AFT and AHe ages across the plateau and through time (Fig. 5) using the same model configuration as before (Fig. 4). To do this, we solved the 1D conduction/advection equation at each point to predict thermal histories, from which ages are then computed. For this, we use the erosion-rate history predicted by Fastscape. Our predictions exclude radiogenic heating effects, leading to an initially linear conductive steady-state geothermal gradient—a standard practice when predicting ages for relatively low-temperature systems such as AHe and AFT that are not very sensitive to the curvature of the geotherm. The ages are predicted from the thermal histories, using the same algorithms as in the Pecube software^99,100^, based on solving the solid-state diffusion equation in 1D inside a grain of given size assuming a cylindrical geometry for AHe using the algorithm in ref. ^101^ and on an annealing algorithm using the parameters in ref. ^102^ for AFT. Here the effects of radiation damage are omitted, potentially changing the absolute age values of the predicted ages, but—notably—not the first-order patterns of interest here. Our models cover a 50-Myr period, with an extra 50 Ma added to computed ages to account for an assumed ‘quiet’ post-uplift phase since the late Cretaceous, consistent with present-day low erosion rates (as measured by cosmogenic methods, for example, ref. ^103^), even along the escarpment. Note that, east of the last position of the mantle convective instability in our model (Fig. 4), the ages return to their assumed ‘un-reset’ value, that is, ≥100 Ma (Fig. 5).

### Testing broader applicability

To explore the broader applicability of our model, we consider the Namibian margin as well as the escarpments and associated plateaus in Eastern Brazil and the Western Ghats (Fig. 1 and Extended Data Fig. 1). First, rifting commenced along the Namibian margin between 145 and 139 Ma, followed by continental break-up occurring from about 116 to 113 Ma (refs. ^54,104^). Indeed, inverse modelling of AHe dates from the Namibian margin, combined with AFT data, reveals an early abrupt cooling phase (10 °C Myr^−1^) from 130 to 100 Ma (ref. ^105^)—probably related to rift shoulder uplift and escarpment retreat—which mirrors South African and Brazilian patterns during continental break-up (see main text). Importantly, offshore AFT analysis and sediment accumulation records overlapping this cooling phase (from 110–80 Ma) indicate the subsequent removal of Precambrian material from the cratonic interior^106^, supporting a spatiotemporal shift inland in the locus of uplift and denudation. Consistent with this, large-scale denudation (that is, 2–3 km in total from 140 to 70 Ma) extended several hundred kilometres inboard of the escarpment^107^, sampling Precambrian lithologies from the Damara Belt and Otavi Group. More than half of this denudation occurred after rifting, closely matching observations in South Africa (Fig. 3) and consistent with our landscape-evolution models (Fig. 4b). Thermal history models across this broader region indicate near-continuous exhumation since the Upper Cretaceous^108^, suggestive of a continuing process. This is again consistent with the concept of sequential migration, with uplift and denudation occurring successively further inland. We can test this concept by considering the distance from the site of exhumation to the nearest COB. Our model (Figs. 2 and 4) predicts substantial exhumation in the interior plateau regions of Southern Namibia around 65–70 Ma. Supporting this, Gallagher and Brown^107^ infer 1–2-km denudation in this region during this period, whereas Wildman et al.^106^ report a Damara uplift phase within a similar time frame from 65 to 60 Ma. Although the specifics of the migration of uplift and denudation into the continental interior remains uncertain given available data, collectively, these observations support a common mechanism across the wider Southern African region associated with rifting and break-up.

Like the Namibian margins, Eastern Brazil underwent rifting at about 139 Ma and continental break-up at around 118 Ma (ref. ^54^). To examine the applicability of our model to Eastern Brazil, and noting a paucity of thermal history analyses in the continental interior, we initially focus on the analysis in ref. ^57^, which extends nearly 1,000 km inland, enabling a comparison with trends on the Southern African plateau. We also consider AFT and AHe ages from across Eastern Brazil using a recent compilation of 1,248 ages^85^, predominantly featuring young ages in lowlands and near escarpments. As predicted by our landscape-evolution models (Figs. 4b and 5), we observe younger ages near the escarpments owing to high magnitudes of total erosion there (Extended Data Fig. 10a). We constructed a profile of AHe and AFT ages in the section in which AHe ages extend furthest inland and observed that, although scattered, the AHe ages decrease nearer the escarpments and decrease with distance into the interior of the plateau (Extended Data Fig. 10c), similar to the observed trend in Southern Africa (Extended Data Fig. 9b).

Our landscape models predict that AFT ages may be much older on the plateau (Fig. 5). This is observed in the São Francisco Craton and marginal orogens, in which old ages (predominantly 350–280 Ma) relate to the Gondwanide orogeny (Extended Data Fig. 10a,b). It is perhaps not surprising that the AFT ages are barely, if at all, reset in the continental interior, in contrast to AHe (Extended Data Fig. 10a,c). This is because the closure temperature for the AFT system is higher than that for AHe (Fig. 5) and considering that the total exhumation across the highlands is low, at about 1.0–1.5 km (Extended Data Fig. 10e). Further, in a study of Northeastern Brazil, Sacek et al.^109^ recognized that the high variability in erodibility, and consequently AFT ages, may result from the formation of duricrust layers or cangas, which could lead to highly variable erosion rates, even under a smoothly varying uplift rate. This factor is probably important in the studied region as well.

Nonetheless, many interior regions have experienced some degree of Cenozoic unroofing. Harman et al.^56^ identified early cooling around 130 Ma at the São Francisco Craton margins during rifting, followed by a later event (circa 60–80 Ma) affecting the cratonic interior of Northeastern Brazil, analogous to the uplift and denudation history of Namibia. Although Harman et al.^56^ attribute this to intracratonic basin inversion (considering the time separation, these authors rule out a role for rifting), the observed uplift of interior regions at these times is consistent with our model predictions (Extended Data Fig. 10c–e), thus offering a new explanation. Extrapolating from Southern African trends (that is, migration rates of the erosional wave; Fig. 3d), our model would predict exhumation onset in these more distal interior regions around 80–25 Ma, as well as recent and continuing exhumation. The observed cooling from about 80 Ma to the present day supports this prediction^26,30,56,57,85,110,111^. Although we do not suggest that all cooling in this region—characterized by several geotectonic provinces, cratonic fragments and tectonic weaknesses (for example, ref. ^30^)—is exclusively linked to mantle instabilities tied to rifting, our model is well supported by existing thermochronological data. The predictions from our model and thermochronology should prompt and inform further measurements, especially AHe analyses, in relatively understudied inland/highland regions of Eastern Brazil.

Testing our model in the Western Ghats is more challenging; however, available data cannot definitively rule it out. The escarpment is associated with the rifting between Madagascar and India, which started around 100 Ma, followed by continental break-up between 86 and 82 Ma (refs. ^54,104^) (Extended Data Fig. 1). Another major escarpment along the eastern passive margin of India^112^ is associated with the earlier separation of India and Antarctica around 125–120 Ma (ref. ^54^). Further rifting and break-up between India and the Seychelles Microcontinent occurred at roughly 66 Ma. Because peninsular India is narrow compared with internally drained continental hinterlands such as Southern Africa and Brazil^113^, in the context of our model (Figs. 2 and 4), interior hinterland regions are expected to exhibit interference patterns in exhumation related to the activity of diachronous rift systems. This issue greatly complicates the identification of drivers of post-rift uplift in the continental hinterlands. During the Cretaceous and Cenozoic, regions inboard of the Western Ghats escarpment experienced high denudation rates (ranging from 50 to 150 m Myr^−1^, depending on the AFT parameters used) associated with syn-rift and post-rift phases^113^, consistent with model expectations (Fig. 4c). Although AFT-derived and mass-balance-derived denudation rates^113^ favour a peak in denudation intensity coinciding with Seychelles rifting^114,115^, it is hard to exclude the possibility of this signal constituting a lagged response to the Madagascar break-up, with post-rift exhumation targeting interior regions. Future studies can apply thermochronology to test these models.

Further references are cited in the extended data^81,116,117^.

## Online content

Any methods, additional references, Nature Portfolio reporting summaries, source data, extended data, supplementary information, acknowledgements, peer review information; details of author contributions and competing interests; and statements of data and code availability are available at 10.1038/s41586-024-07717-1.

### Supplementary information

Supplementary Video 1 Video of ASPECT thermomechanical simulations of continental break-up in which extension velocities are distributed symmetrically at both side boundaries. The process of sequential delamination occurs as described in the reference model. The logarithmic strain rates and temperatures in the asthenosphere are shown (see key).

Supplementary Video 2 Video of ASPECT thermomechanical simulations of continental break-up under a slow extension regime (5 mm year^−1^) showing the generation and sequential propagation of Rayleigh–Taylor instabilities along the lithospheric keel. The logarithmic strain rates and temperatures in the asthenosphere are shown (see key).

Supplementary Video 3 Video of ASPECT thermomechanical simulations of continental break-up under a fast extension regime (20 mm year^−1^) showing the generation and sequential propagation of Rayleigh–Taylor instabilities along the lithospheric keel. The logarithmic strain rates and temperatures in the asthenosphere are shown (see key).

Supplementary Video 4 Video of ASPECT thermomechanical simulations of continental break-up in which the vertical extent of the model domain is 410 km instead of 300 km (reference model). The process of sequential delamination occurs as described in the reference model. Migration rates differ slightly from the reference model but are in full agreement with observational constraints (see Methods). The logarithmic strain rates and temperatures in the asthenosphere are shown (see key).

### Source data

Source Data Fig. 1

Source Data Fig. 3

Source Data Fig. 4

Source Data Fig. 5

Source Data Extended Data Table 3

## Figures and Tables

**Extended Data Table 1:**
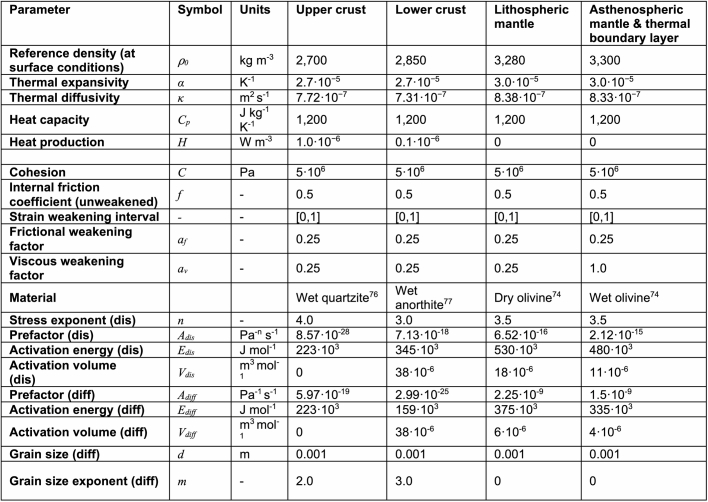
Model parameters used in ASPECT thermomechanical simulations (see Methods for details)

diff, diffusion creep (some values are from refs. ^74,76,77^); dis, dislocation creep.

**Extended Data Table 2:**
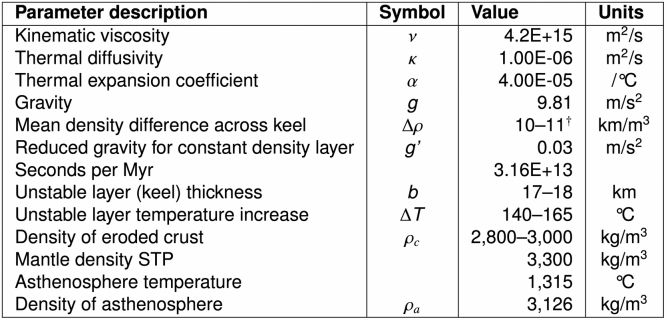
Parameters used in the analytical models of uplift and denudation

The models investigate how the delamination of an unstable keel layer (see ref. ^11^) affects the uplift of Earth’s surface and the extent of maximum denudation (see main text and Methods for details). ^†^Note that the given density difference across the keel assumes a density contrast between cold lithosphere in the TBL and hot asthenosphere and does not consider compositional changes.

**Extended Data Table 3:**
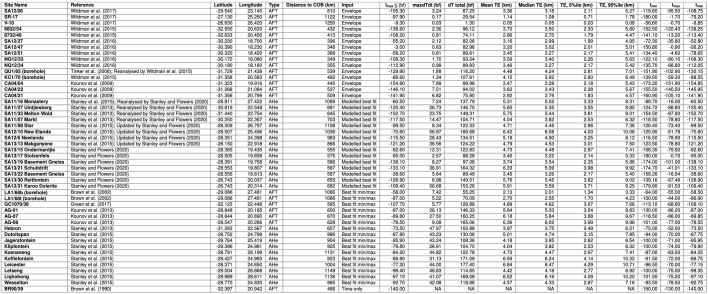
Exhumation characteristics of thermochronology sites spanning the Central Plateau of Southern Africa

Thermal history models of the sites are from the references provided^5,25,29,31,44,48,50,53,55,81,82,117^. Note that maxd*T*d*t* (bf) is the maximum rate of temperature drop in °C over a 2-Myr rolling window using the best fit (bf) curve and ${t}_{\max }\frac{{\rm{d}}T}{{\rm{d}}t}$ denotes the timing of maxd*T*d*t* (bf) in Ma, presented as a negative value. TE refers to total exhumation (note that these values are sampled to account for uncertainty in geothermal gradients used; see Methods). *t*_min_, *t*_mid_ and *t*_max_ are the minimum, midpoint and maximum times of maximum cooling accounting for available (published) model data and uncertainty estimates. More details are provided in Methods.

Source Data

## Data Availability

The thermochronological data used in our analysis were accessed from published studies, which are listed in Extended Data Table 3 and Extended Data Fig. 9 (southern Africa) and in Extended Data Fig. 10 (Brazil). The data generated in this study are provided in Extended Data Table 3 and are available as Source Data files in the online version of this article. Source data are provided with this paper.

## Code availability

The geodynamic modelling software ASPECT can be obtained from https://github.com/geodynamics/aspect. The input file, custom source code and ASPECT installation details for the thermomechanical simulations are available from the Zenodo repository at 10.5281/zenodo.10692548 (ref. ^118^). The Fastscape landscape-evolution model can be accessed and downloaded from https://github.com/fastscape-lem/fastscape and is fully documented at https://fastscape.readthedocs.io/en/latest/.
